# Novel pharmacological actions of trequinsin hydrochloride improve human sperm cell motility and function

**DOI:** 10.1111/bph.14814

**Published:** 2019-12-11

**Authors:** Rachel C. McBrinn, Joanna Fraser, Anthony G. Hope, David W. Gray, Christopher L.R. Barratt, Sarah J. Martins da Silva, Sean G. Brown

**Affiliations:** ^1^ School of Science, Engineering and Technology Abertay University Dundee UK; ^2^ Drug Discovery Unit, School of Life Sciences University of Dundee Dundee UK; ^3^ Reproductive and Developmental Biology, School of Medicine, Ninewells Hospital and Medical School University of Dundee Dundee UK

## Abstract

**Background and Purpose:**

Asthenozoospermia is a leading cause of male infertility, but development of pharmacological agents to improve sperm motility is hindered by the lack of effective screening platforms and knowledge of suitable molecular targets. We have demonstrated that a high‐throughput screening (HTS) strategy and established in vitro tests can identify and characterise compounds that improve sperm motility. Here, we applied HTS to identify new compounds from a novel small molecule library that increase intracellular calcium ([Ca^2+^]_i_), promote human sperm cell motility, and systematically determine the mechanism of action.

**Experimental Approach:**

A validated HTS fluorometric [Ca^2+^]_i_ assay was used to screen an in‐house library of compounds. Trequinsin hydrochloride (a PDE3 inhibitor) was selected for detailed molecular (plate reader assays, electrophysiology, and cyclic nucleotide measurement) and functional (motility and acrosome reaction) testing in sperm from healthy volunteer donors and, where possible, patients.

**Key Results:**

Fluorometric assays identified trequinsin as an efficacious agonist of [Ca^2+^]_i_, although less potent than progesterone. Functionally, trequinsin significantly increased cell hyperactivation and penetration into viscous medium in all donor sperm samples and cell hyperactivation in 22/25 (88%) patient sperm samples. Trequinsin‐induced [Ca^2+^]_i_ responses were cross‐desensitised consistently by PGE_1_ but not progesterone. Whole‐cell patch clamp electrophysiology confirmed that trequinsin activated CatSper and partly inhibited potassium channel activity. Trequinsin also increased intracellular cGMP.

**Conclusion and Implications:**

Trequinsin exhibits a novel pharmacological profile in human sperm and may be a suitable lead compound for the development of new agents to improve patient sperm function and fertilisation potential.

**What is already known**

- There is an unmet clinical need for compounds to treat asthenozoospermia (poor sperm motility).

**What this study adds**

- Trequinsin hydrochloride raised intracellular calcium and cyclic GMP in human sperm and improved motility.

**What is the clinical significance**

- Trequinsin hydrochloride has clinically relevant positive effects on human sperm motility.
- Thus, trequinsin hydrochloride has the potential to be a novel treatment for male infertility.

**Abbreviations**
[Ca^2+^]_i_: intracellular calcium
ACU: Assisted Conception Unit
CASA: computer‐assisted sperm analysis
CM: capacitating media
DGC: density gradient centrifugation
Gm: membrane conductance
HA: hyperactivated motility
HTS: high‐throughput screening
ICSI: intracytoplasmic sperm injection
NCM: non‐capacitating media
PDE3i: PDE3 inhibitor
PM: progressive motility
pHi: intracellular pH
sEBSS: supplemented Earls buffered salt solution
TM: total motility
VCL: curvilinear velocity

## 1 INTRODUCTION

Asthenozoospermia (low sperm motility) has been reported as the leading cause of male infertility (Kumar & Singh, 2015). Intracytoplasmic sperm injection (ICSI) is the most common and successful treatment for male infertility. While it is a pragmatic solution, it involves invasive treatment of the female partner and bypasses all natural sperm selection processes. There are concerns that ICSI may be associated with long‐term health issues for the children born, particularly in cases where the spermatozoa are predominately immotile and do not have the capacity to fertilise under natural conditions (Esteves, Roque, Bedoschi, Haahr, & Humaidan, 2018; Hanevik, Hessen, Sunde, & Breivik, 2016). Therefore, the development of novel direct treatments for male infertility is desirable, although this represents a significant challenge because of the limited understanding of the regulation of normal and dysfunctional sperm (Barratt et al., 2017).

Intracellular calcium concentration ([Ca^2+^]_i_) is an established regulator of sperm function, and a wealth of evidence suggests that the principal cation channel in sperm (http://www.guidetopharmacology.org/GRAC/FamilyDisplayForward?familyId=70) influences sperm function and fertilisation potential through regulation of extracellular calcium influx (Singh & Rajender, 2015; Strünker et al., 2011; Tamburrino et al., 2014; Williams et al., 2015). CatSper is confined to the principal piece of the flagellum and is modulated by intracellular pH (pHi) and membrane potential. It is sensitive to http://www.guidetopharmacology.org/GRAC/LigandDisplayForward?ligandId=2377 (Lishko, Botchkina, & Kirichok, 2011; Strünker et al., 2011), which stimulates cell penetration into a viscous medium (used as an in vitro model for regions of the female reproductive tract; Alasmari et al., 2013; Barratt & Publicover, 2012). [Ca^2+^]_i_ also plays a significant role in the regulation of soluble cyclases that drive the production of cyclic nucleotides. These key secondary messengers have been shown to be fundamental for human sperm cell motility, cell capacitation, and acrosome reaction. Cyclic nucleotides are actively enzymatically degraded by http://www.guidetopharmacology.org/GRAC/FamilyDisplayForward?familyId=260, and PDE inhibitors can positively affect sperm cell motility and function (Maréchal et al., 2017; Tardif et al., 2014; Willipinski‐Stapelfeldt et al., 2004).

Identifying CatSper agonists to improve sperm motility and function is a logical approach to drug discovery for male infertility. We have previously described the development of a high‐throughput screening (HTS) system to identify compounds that increase [Ca^2+^]_i_ and thereafter have assessed the functional consequence of in vitro application of two compounds (Martins da Silva et al., 2017). However, sperm motility is multiform and adaptive, and not every patient sample responded to treatment in vitro. As such, there remains a clear need to continue to identify potential therapeutic compounds.

In this study, we hypothesised that novel CatSper agonists could be identified by screening a library of small molecules with defined molecular targets (chemogenomic library). This library was assembled from well‐characterised, commercially available ligands (Tocris) for a range of validated drug targets including enzymes, receptors, and transporters. We demonstrate that https://www.guidetopharmacology.org/GRAC/LigandDisplayForward?ligandId=10436, a http://www.guidetopharmacology.org/GRAC/ObjectDisplayForward?objectId=1298 inhibitor (PDE3i; Degerman, Belfrage, & Manganiello, 1997; Lal, Dohadwalla, Dadkar, D'Sa, & de Souza, 1984), is highly effective at inducing an increase in [Ca^2+^]_i_, which corresponded with improved sperm motility. Detailed characterisation of the mechanism of action of trequinsin suggests that these effects are achieved through complex and novel pharmacological activities in human spermatozoa.

**Figure 1:**
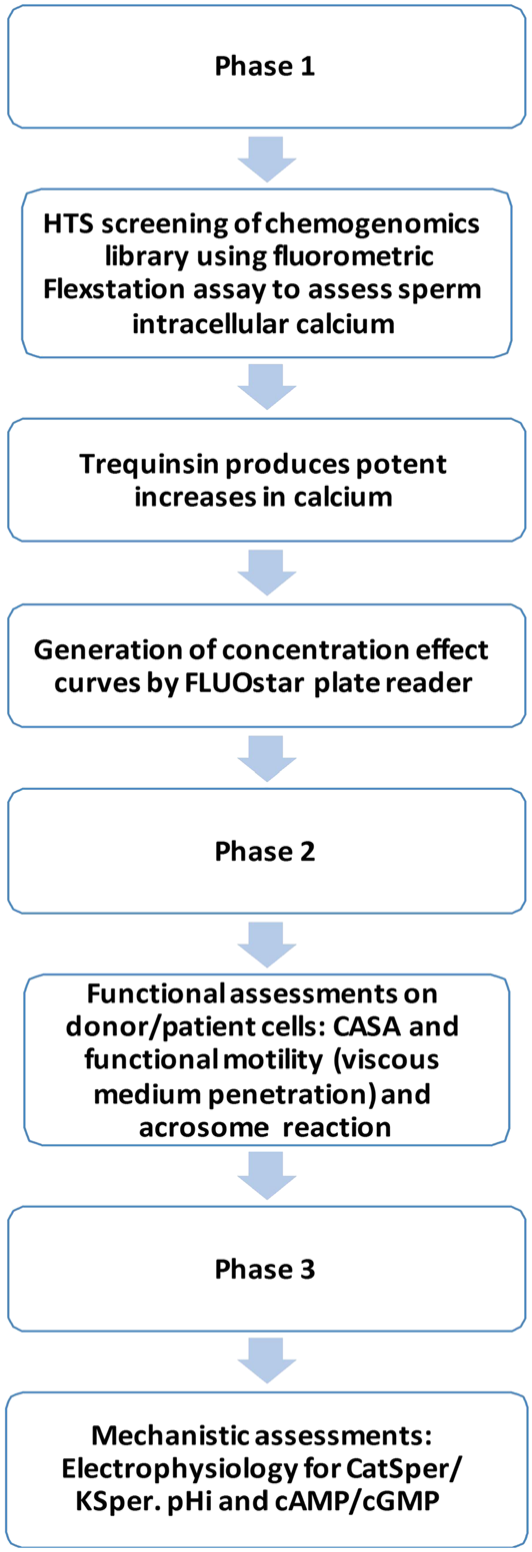
Experimental plan. Systematic functional and mechanistic screening strategy for the identification of the molecular and functional effects of trequinsin

The study aimed to investigate hit compounds from a chemogenomic drug library screen for effects on sperm motility and to determine the mechanism responsible. This was achieved in three phases. Phase 1 employed HTS of compounds for their ability to increase sperm [Ca^2+^]_i_ relative to a saturating concentration of progesterone (P4). Phase 2 involved detailed sperm function tests, and Phase 3 involved molecular analysis of trequinsin hydrochloride, which was selected due to its high efficacy in Phase 1 and its purported PDE3i activity. An outline of the experimental approach is shown in Figure 1.

## 2 METHODS

### 2.1 Ethical approval

Study approval was in accordance with the Human Fertilisation and Embryology Authority Code of Practice (version 8) and local ethical approval (13/ES/0091) from East of Scotland Research Ethics Service REC 1. Following informed consent, samples for research were obtained from patients undergoing investigation and treatment at the Assisted Conception Unit (ACU), Ninewells Hospital, Dundee, and that were surplus to clinical requirement. Samples from healthy volunteer research donors with normal sperm motility parameters in agreement with World Health Organization 2010 criteria (Cooper et al., 2010) were used in this study under the same ethical approval. All obtained samples for research were analysed in line with suggested guidance for human semen studies where appropriate (Björndahl, Barratt, Mortimer, & Jouannet, 2015).

### 2.2 Preparation of donor and patient sperm samples

All donors and patients adhered to an abstinence period of 2–5 days before sample collection by masturbation into a sterile plastic container. The sample was placed in a 37°C incubator for 30 min to allow liquefaction. Semen samples from patients were categorised according to World Health Organization guidelines (Cooper et al., 2010).

Donor and andrology semen samples were prepared by density gradient centrifugation (DGC) as described by Martins da Silva et al. (2017). Solutions for the manufacturing of non‐capacitating media (NCM), capacitating media (CM), and density gradient solutions can be found in Appendix S1. Preparation of capacitated patient sperm was carried out by the ACU using commercial media from PureSperm™ (Nidacon, Mölndal, Sweden) and Quinn's Advantage Medium with HEPES (SAGE In‐Vitro Fertilization; Pasadena, CA, USA; Brown et al., 2016).

### 2.3 Chemogenomics library high‐throughput screen

Dundee University Drug Discovery Unit in‐house chemogenomics library was screened for compounds that increase [Ca^2+^]_i_ in human sperm. The compound library is composed of a set of 223 commercially available small molecules and drugs (Tocris), each with a well‐defined mechanism of action, potency at the primary target, and selectivity. The compounds were selected as representative ligands for a diverse range of drug targets including enzymes, GPCRs, ion channels, and transporters. The compound library was initially screened on a single 384 well assay plate, at a single concentration of 40 μM. HTS and data analysis were performed as previously described (Martins da Silva et al., 2017). In brief, spermatozoa from two to four different donors were pooled together after preparation by DGC, diluted to a density of 2.2 × 10^7^·ml^−1^ in Flexstation assay buffer (1 X HBSS [Invitrogen], 20‐mM HEPES, 0.5‐mM probenecid, pH 7.4), and incubated for 60 min (37°C) with 2 x Calcium 3 dye (Molecular Devices). Spermatozoa were washed following incubation, resuspended in Flexstation assay buffer, and plated in 384 well clear bottom, black well assay plates (Greiner Bio One) at a density of 2.5 × 10^5^ cells/50 μl per well. [Ca^2+^]_i_ was measured using a Flexstation 3 (Molecular Devices). Baseline calcium‐dependent fluorescence (excitation wavelength = 485 nm, emission wavelength = 525 nm, and cut‐off = 515 nm) was measured for 18 s; 12.5 μl of each test compound was transferred to the assay plate using an internal 16‐channel robotic pipette head, and the resulting change in fluorescence was monitored for a further 82 s. Follow‐up assays were performed to determine the potency of hit compounds. All assay plates in the screen were subject to quality control analysis.

Preliminary analysis of all HTS primary and potency raw data was performed using the AUC function within the SoftMax Pro analysis software (http://www.moleculardevices.com/systems/microplate-readers/softmax-pro-data-acquisition-and-analysis-software, RRID:SCR_014240) to quantitate agonist‐evoked fluorescence as previously described. Data were exported as a text file for further data processing and analysis in Activity Base version 7.3.1.4 (IDBS), and the percentage effect for each compound was normalised to the paired positive control (10‐μM P4). Compounds were pragmatically classified on the basis of calcium fluorescence elicited and designated as low responder (blue 20–49%), mild responder (orange 50–89%), and high responder (green 90–120%) relative to progesterone (Table 1).

**Table 1:** Summary of [Ca^2+^]_i_ elevating compounds identified in screen of the chemogenomics library

Compound name	Primary action (Tocris)	Percentage increase in fluorescence in sperm
Zaprinast	PDE5/6/9/11 inhibitor	23
SB 218078	Inhibitor of checkpoint kinase 1 (Chk1)	25
RO‐3	Selective P2X3 and P2X2/3 antagonist	26
GP 1a	Highly selective CB_2_ receptor agonist	32
NNC 55‐0396 dihydrochloride	Highly selective Ca^2+^ channel blocker (T‐type)	32
EHT 1864	Potent inhibitor of Rac family GTPases	32
SD 208	Potent ATP‐competitive TGFRI inhibitor	34
SANT‐2	Inhibitor of hedgehog (Hh) signalling; antagonises smoothened activity	36
Repaglinide	KATP channel blocker	38
EO 1428	Selective inhibitor of p38α and p38 α_2_	39
BI 78D3	Selective, competitive JNK inhibitor	45
IKK 16	Selective inhibitor of IKK	47
BRL 50481	Selective PDE7 inhibitor	49
Calcipotriol	Vitamin D3 analog	51
AS 1949490	SH2 domain‐containing inositol 5′‐phosphatase 2 (SHIP2) inhibitor	54
U 89843A	Positive allosteric modulator of GABA_A_ receptors	57
SANT‐1	Inhibitor of hedgehog (Hh) signalling; antagonises smoothened activity	57
Ciglitazone	Selective PPAR agonist	64
UK 78282 hydrochloride	Blocker of KV1.3 and KV1.4 channels	66
GW 9508	Potent and selective FFA1 (GPR40) agonist	67
FPL 64176	Potent activator of Ca^2+^ channels (L‐type)	75
NVP 231	Potent, selective, and reversible CerK inhibitor	77
Y 29794 oxalate	Prolyl endopeptidase inhibitor	88
Trequinsin hydrochloride	Ultrapotent inhibitor of PDE3	91
Lylamine hydrochloride	CB_1_ receptor agonist	108
PHA 665752	Potent and selective MET inhibitor	111
JX 401	Potent, reversible p38α inhibitor	115

*Note.* Twenty‐seven U.S. Food and Drug Administration‐approved active compounds were identified from the DDU Chemogenomics library screen following Flexstation assay testing, and categorised based on their ability to increase [Ca^2+^]_i_ (low to high percentage increase relative to 10‐μM progesterone [positive control]). Trequinsin hydrochloride was selected for this study as it was highly efficacious and a PDE inhibitor (the compound library screen of all 223 commercially available small molecules and drugs [Ca^2+^]_i_ is shown in Figure S12).

### 2.4 Motility assessment: Computer‐assisted semen analysis

Prepared spermatozoa were incubated for 3 hr at 37°C in CM or NCM as appropriate and then mixed with DMSO (vehicle control, 1% final concentration) or trequinsin (10‐μM final concentration; Tocris Bioscience, Abingdon, UK). Sperm cells were incubated for 20 min; then motility was assessed (using four‐chamber 20‐μM deep slides; Vitrolife, Sweden). At least 200 sperm cells were analysed per chamber per condition for each motility parameter (Tardif et al., 2014). Motility readings were recorded over a 2‐hr period (0, 20, 40, 60, 90, and 120 min) for donor and patient samples using computer‐assisted sperm analysis (CASA; CEROS machine [version 12], Hamilton Thorne Research, Beverly, MA, USA). Parameters measured included progressive motility (PM), total motility (TM), and hyperactivated motility (HA). Proprietary algorithms on the CASA determined the percentage of cells displaying HA automatically. Specifically, a subpopulation sperm displaying curvilinear velocity (VCL) ≥150 μm·s^−1^, linearity <50%, and amplitude of lateral head displacement ≥7 μm of algorithms were designated as hyperactive (Mortimer & Mortimer, 2013).

### 2.5 Motility assessment: Sperm penetration test

Sperm penetration test was conducted using Kremer tubes (0.4 × 4 mm of internal diameter; CM Scientific Ltd, New Jersey, USA) placed into Eppendorf tubes containing approximately 1 × 10^5^ spermatozoa in CM at 37°C, 5% CO_2_ for 1 hr (Martins da Silva et al., 2017). The number of spermatozoa was counted manually at 1 and 2 cm and compared between control (1% DMSO), 10‐μM trequinsin, 3.6‐μM progesterone, and 500‐μM http://www.guidetopharmacology.org/GRAC/LigandDisplayForward?ligandId=388, a non‐specific agonist for sperm motility used as a PDEi positive control (Strünker et al., 2011). Data were normalised to paired controls and expressed as a penetration index, that is, number of spermatozoa observed with treatment/number of spermatozoa without treatment (control; Ivic et al., 2002; Martins da Silva et al., 2017).

### 2.6 Flow cytometry analysis

Following 3‐hr incubation in capacitating conditions, two aliquots containing 2 × 10^−6^ sperm were centrifuged at 0.3 x *g* for 5 min. The supernatant was removed, and the pellets were resuspended in a staining solution containing (final concentrations): 10 μg·ml^−1^ of Alexa Fluor™ 647 conjugated peanut agglutinin (PNA‐647, Life Technologies Ltd, Paisley, UK) and 0.8 μg·ml^−1^ of propidium iodide (Life Technologies Ltd) in supplemented Earls buffered salt solution (sEBSS). Control‐treated (1% DMSO) and trequinsin‐treated (10 μM) sperm were incubated at 37°C/5% CO_2_ for 20 min prior to flow cytometry analysis. Paired positive controls were conducted within each experiment using control cells treated with the calcium ionophore A21387 (10 μM) and Triton X‐100 (0.1%) to induce the acrosome reaction and cell membrane damage, respectively.

The effect of trequinsin on acrosome reaction and membrane integrity was assessed using an Intellicyt iQue Screener equipped with a 488‐nm laser. In accordance with Intellicyt guidelines, emission of fluorescence was detected using fluorescence detector 3 (670‐nm LP filter) and 4 (675/25 nm) for propidium iodide and PNA‐647, respectively. Forward scatter and side scatter fluorescence data were recorded from a minimum of 10,000 events per condition. Threshold levels were selected to exclude cellular debris, and the gates to discriminate between live/dead and acrosome‐reacted/non‐reacted were set using the positive control samples. Data were analysed using Intellicyt's proprietary Forecyt software.

### 2.7 [Ca^2+^]_i_ fluorescence measurements

After incubation for 3 hr in CM or NCM, approximately 3 × 10^−6^ per ml^−1^ of spermatozoa were incubated with 4.5 μM of FLUO‐4 AM, (Thermo Fisher Scientific, Oregon, USA) for 20 min at 37°C, 5% CO_2_ before centrifugation at 500 *g* for 3 min. The supernatant was removed, and the pellet was resuspended in sEBSS (Supporting Information). Fluorescence measurements were carried out on a FLUOstar Omega reader (BMG Labtech, Offenburg, Germany) at 37°C; 3 × 10^5^ cells were imaged per well (Martins da Silva et al., 2017; Tardif et al., 2014). To construct the trequinsin dose–response curve, the trequinsin data were normalised to paired [Ca^2+^]_i_ response evoked by 3.4‐μM progesterone (to control for unwanted sources of variation).

Desensitisation experiments were carried in accordance with an established methodology (Brenker et al., 2018; Schaefer, Hofmann, Schultz, & Gudermann, 1998; Strünker et al., 2011). The first compound addition was added after 1‐min recording of baseline fluorescence, followed by addition of the second compound after 5 min. Control experiments were conducted to demonstrate that progesterone and https://www.guidetopharmacology.org/GRAC/LigandDisplayForward?ligandId=1882 do not cross‐desensitise. Control experiments to demonstrate desensitisation, involved either addition of progesterone followed by http://www.guidetopharmacology.org/GRAC/LigandDisplayForward?ligandId=5104 or addition of PGE_1_ followed by http://www.guidetopharmacology.org/GRAC/LigandDisplayForward?ligandId=1883. The protocol used to assess the mode of action of trequinsin was similar. Cells were first challenged with either progesterone or PGE_1_ followed, after 5 min, by trequinsin. Readings from an additional time control well (baseline) were taken as were readings from a well that was exposed to a single agonist at the time point that matched the time point of addition of the second agonist in the desensitisation experiments. All compounds were used at a final concentration of 10 μM.

### 2.8 Measurement of pHi

After 3 hr in CM, spermatozoa (4 × 10^−6^ per ml^−1^) were incubated with 2‐μM 2′,7′‐bis(2‐carboxyethyl)‐5,6‐carboxyfluorescein (ThermoFisher, Paisley, UK) for 30 min at 37°C. The cells were centrifuged for 3 min at 500 x *g*, the supernatant was removed, and the cells were then resuspended in sEBSS. A FLUOstar Omega reader (BMG Labtech) was used to detect the emitted fluorescence (excitation wavelength ratio of 440/490 nm and emission wavelength of 530 nm). Cell calibration was achieved following cell lysis by the addition of 1% Triton X‐100, a reading was taken from each well, and a calibration curve was constructed using 1‐M HCl and 1‐M NaOH. Fluorescence measurements for control (cells +1% DMSO) and trequinsin (10 μM) were recorded, as well as ammonium chloride (NH_4_Cl), which was used as a positive control (10‐mM final concentration).

### 2.9 Electrophysiology

The effect of trequinsin on individual sperm plasma membrane ion channels was investigated using whole‐cell patch clamp electrophysiology (Brown et al., 2016). Sperm were allowed to settle on a glass coverslip prior to being placed in the recording chamber that was perfused with standard extracellular solution (Supplementary Solutions in the Supporting Information). Gigaseals were achieved between sperm midpiece and high resistance (8–12 MΩ) borosilicate glass pipettes filled with either quasi‐physiological standard intracellular solution or Cs^+^‐based divalent‐free intracellular solution to study membrane slope conductance (Gm) that is predominantly carried by K^+^ ions (Brown et al., 2016) and CatSper channels, respectively (Supporting Information). Transition to whole‐cell configuration was achieved by applying brief suction. To study outward membrane conductance, a depolarising ramp protocol was imposed (−92 to 68 mV) over 2,500 ms, and membrane potential was held at −92 mV between test pulses. The effect of trequinsin on reversal potential and membrane slope conductance of outward currents was assessed by regression analysis over the voltage range where membrane current crosses the *x* axis (*I* = 0) and outward current from 20 to 68 mV, respectively (Brown et al., 2016).

After achieving the whole‐cell configuration, monovalent CatSper currents were recorded by superfusing sperm with Cs^+^‐based divalent‐free bath solution (Supplementary Solutions in the Supporting Information). Currents were evoked by a ramp protocol (−80 to 80 mV over 1 s). Membrane potential was held at 0 mV between ramps. Data were sampled at 2 kHz and filtered at 1 kHz (PClamp 10 software, Axon Instruments, USA). The post‐recording analysis was conducted as described previously to adjust for liquid junction potential and normalise for cell size (Brown et al., 2016).

### 2.10 Detection of cyclic nucleotides by reversed‐phase HPLC

#### 2.10.1 HPLC sample preparation

After 3‐hr incubation in CM, 9 × 10^−6^ per ml^−1^ of spermatozoa were treated with 1% DMSO (vehicle control), 10‐μM trequinsin, or 500‐μM IBMX (positive control) and incubated for a further 20 min. The samples were centrifuged (5 min, 300 x *g*), the supernatant was removed, and the pellet was resuspended in 0.5 ml of 100‐mM sodium acetate (pH 4), sonicated for 1 min in a water bath, briefly vortexed, and centrifuged again (5 min, 3,000 *g*). The supernatant was removed and placed in a fresh Eppendorf, snap frozen in liquid nitrogen, and stored on dry ice until solid phase extraction.

#### 2.10.2 Solid phase extraction

Cyclic nucleotides were extracted using Strata™‐X‐AW (Phenomenex, Cheshire, UK) 33‐μM polymeric weak anion solid phase extraction cartridges. Cartridges were pretreated with 1:1 of 100‐mM sodium acetate and water (final pH 4), conditioned with 0.5 ml 100% methanol, and equilibrated with 0.5 ml of 100‐mM sodium acetate (final pH 4). The supernatant was then loaded into the cartridge, washed with 0.5 ml of sodium acetate (final pH 4), followed by 0.5 ml of 100% methanol, and dried for 5 min under full vacuum before 0.5 ml of 28–30% (w/v) solution of ammonium hydroxide was added to methanol (95:5) in order to elute the cyclic nucleotides into ice‐cold 1.5‐ml centrifuge tubes. This solution was dried under nitrogen for 30 min and suspended in 0.5 ml of mobile phase (20‐mM potassium phosphate in 100% ultrapure water, 0.1% TFA, and 0.1% ACN, pH 2.8 adjusted with 2.5% phosphoric acid).

#### 2.10.3 Standards and stock solutions

Stock solutions of cyclic nucleotides (http://www.guidetopharmacology.org/GRAC/LigandDisplayForward?ligandId=2352 and http://www.guidetopharmacology.org/GRAC/LigandDisplayForward?ligandId=2347) were prepared to 3 mol·L^−1^ in mobile phase (see Section 2.10.4). From the stock solutions, five 10‐fold serial dilutions were produced to achieve a 6‐point standard curve (peak AUC). This was used for quantification of cyclic nucleotides in sperm samples.

#### 2.10.4 HPLC set‐up

The HPLC system comprised Waters 1525 binary HPLC pump, 2487 dual λ absorbance detector, 717 plus autosampler, and a Synergi™ 4‐μm fusion—RP80A (Phenomenex, Cheshire, UK) C18 analytical column (150 mm × 4.6 mm of internal diameter, 4‐μm particle size). Mobile phase: Isocratic elution of 100% 20‐mM potassium phosphate in ultrapure, filtered and degassed water, with 0.1% TFA and 0.1 ACN, pH 2.8 with 2.5% phosphoric acid. Chromatographic conditions: Flow rate: 1 ml·min^−1^. Injection volume: 200 μl. Detection wavelengths: 255 nm (cGMP) and 256 nm (cAMP).

### 2.11 Data and statistical analysis

The data and statistical analysis comply with the recommendations on experimental design and analysis in pharmacology (Curtis et al., 2018). This research did not include the use of animals. Statistical power analysis was conducted to ensure that the group size was sufficient to measure an effect for each experiment using R pwr package (https://www.r-project.org/, RRID:SCR_001905; pwr.2p.test) and Cohen's effect size analysis (control vs. treatment, sig. level = 0.05, power = 0.8). *N* numbers refer to data from independent samples. Donor samples were allocated randomly by the technical team, and patient samples were provided by the ACU based on consent and recruitment on the day of treatment. In cases of analysis of responses from individual patients, replicates were not possible. Therefore, the analysis was conducted, as indicated in Section 2.12. Blinding of the operator and the data analysis were not undertaken. However, several investigators were used throughout the study to ensure the consistency of observed effects.

A total of 28 donors and 25 patients were included in this study. Statistical comparisons for the effect of trequinsin versus control conditions used paired *t* tests, unpaired *t* tests, or two‐way ANOVA and Sidak's multiple comparison analysis as appropriate using the statistical package GraphPad Prism 7 (La Jolla, CA, USA; http://www.graphpad.com/, RRID:SCR_002798) unless stated otherwise. *P* < .05 as represented by * is considered significant. [Ca^2+^]_i_ and pHi studies were recorded as the percentage change in fluorescence from baseline conditions. For the analysis of individual patient sperm motility results, statistical significance was recorded when the ±*SD* did not overlap for control and treatment conditions (Tardif et al., 2014). HPLC data were extracted using Breeze 2 software and analysed using Microsoft Excel. Results are expressed as pmol per 10^6^ cells.

### 2.12 Nomenclature of targets and ligands

Key protein targets and ligands in this article are hyperlinked to corresponding entries in http://www.guidetopharmacology.org, the common portal for data from the IUPHAR/BPS Guide to PHARMACOLOGY (Harding & Sharman et al., 2018), and are permanently archived in the Concise Guide to PHARMACOLOGY 2017/18 (Alexander, Fabbro et al., 2017; Alexander, Striessnig et al., 2017).

## 3 RESULTS

### 3.1 Phase 1: Drug library screen

Dundee University Drug Discovery Unit chemogenomic library compounds were screened for their ability to evoke an increase in [Ca^2+^]_i_ in capacitated sperm relative to a saturating dose of progesterone (3.6 μM) using a fluorometric HTS assay and Flexstation 3 microplate reader. When tested at a single concentration of 40 μM, we identified 27 putative hits eliciting >23% effect (12.1% hit rate), 23 putative hits with >50% effect (10.3% hit rate), and four putative hits with >90% effect (1.8% hit rate; Table 1). Trequinsin hydrochloride was notable among the compounds eliciting the greatest increase in [Ca^2+^]_i_, as it is a PDE enzyme inhibitor (PDEi) and we have previously shown that similar compounds provide clinically relevant enhancement of sperm motility (Tardif et al., 2014). Trequinsin caused a concentration‐dependent increase in [Ca^2+^]_i_ (EC_50_ = 6.4 μM [95% confidence interval (CI): 4.1–9.9 μM]; Figure S1). The functional and molecular profile of trequinsin was studied in further detail, as presented in this report. The other three compounds eliciting >90% increase in [Ca^2+^]_i_ did not promote motility (as assessed by CASA, data not shown) and were therefore not studied further.

### 3.2 Phase 2: Functional effects of trequinsin

#### 3.2.1 Donor sperm assessment

**Figure 2:**
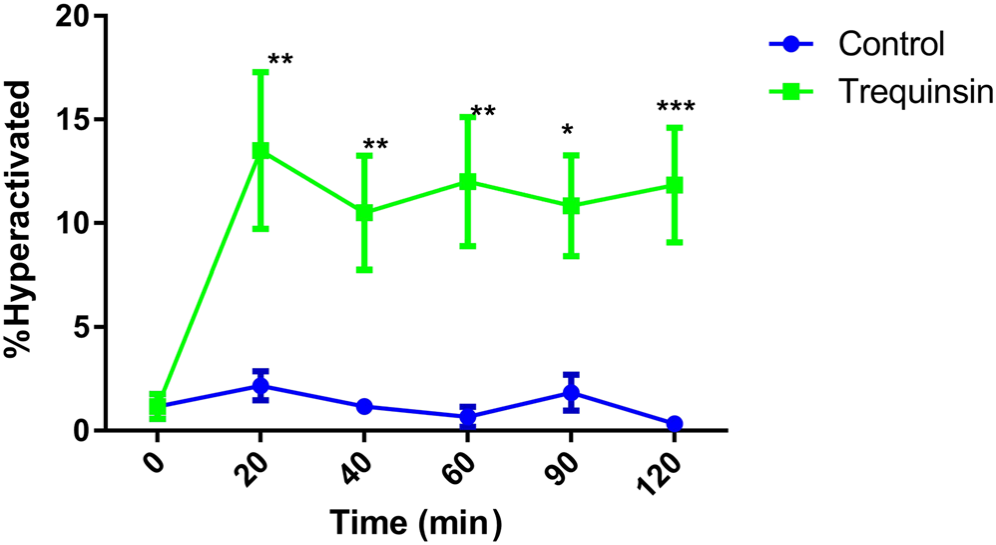
Effect of trequinsin on capacitated donor sperm cell hyperactivation*.* Trequinsin significantly increased HA in all donor cells from the 80% DGC fraction exposed to capacitating conditions. *P<.05, significantly different from start (time 0); two‐way ANOVA with Sidak's multiple comparison analysis. The increase in hyperactivation was sustained for a 2‐hr period after initial exposure. A minimum of 200 cells were counted at each time point. Hyperactivation classified by CASA parameters: VCL >150 μM·s^−1^, linearity <50%, and amplitude of lateral head displacement >7 μM. In the same sample set, %TM and %PM were unaffected by the addition of trequinsin (Figure S3)

**Figure 3:**
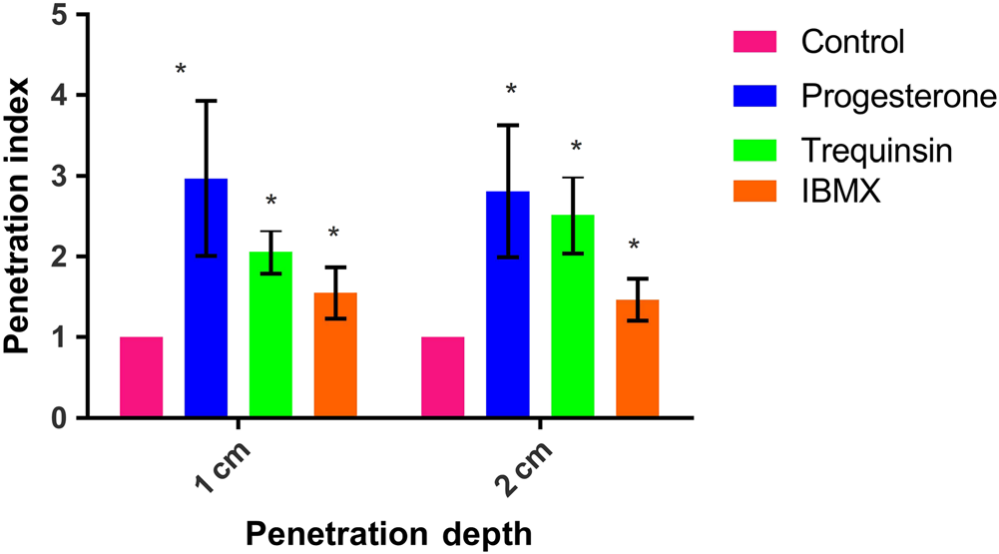
Sperm penetration assay. The ability of trequinsin to stimulate sperm penetration into viscous medium was assessed using capacitated sperm from the 80% DGC fraction (*n* = 5). A significant increase in cell penetration was observed in the presence of trequinsin in comparison to control, but not in comparison to cells stimulated with progesterone (P4). Cell penetration at 1 cm was not significantly different between trequinsin‐ and IBMX‐treated cells. However, trequinsin stimulated a significantly greater cell number to penetrate at 2 cm compared to IBMX. *P<.05, significantly different from control; two‐way ANOVA with Sidak's multiple comparison analysis

It is well accepted that activation of CatSper and elevation of cyclic nucleotides are fundamental for sperm motility and function (Ahmad et al., 2015; Alasmari, Costello, et al., 2013; Lefièvre, de Lamirande, & Gagnon, 2002). Motility and kinematic parameters of capacitated spermatozoa from healthy volunteer donors (80% DGC fraction) after 20‐min exposure to trequinsin were studied using CASA. Trequinsin (0.1–100 μM) had no significant effect on TM or PM (Figure S2). However, a bell‐shaped dose–response curve was obtained for HA (Figure S2). As ≥30 μM had no effect on HA, 10‐μM trequinsin was used in subsequent experiments. Capacitated sperm from the 80% DGC fraction exposed to 10‐μM trequinsin showed no change in TM or PM (Figure S3A,B) over a 2‐hr period. However, the percentage of HA cells sperm was significantly increased (Figure 2). We also assessed the ability of trequinsin to stimulate penetration into viscous medium (Kremer test) as a measure of functional motility in the same spermatozoa population (80%, capacitated). Trequinsin, progesterone, and IBMX all significantly and similarly increased cell penetration into viscous medium at 1 cm. However, trequinsin and progesterone were significantly better than IBMX at stimulating penetration at 2 cm (Figure 3). Trequinsin did not induce premature acrosome reaction in capacitated cells (Figure S4B). In contrast, trequinsin had no effect on the motility parameters of cells from the 80% fraction in non‐capacitating conditions (Figure S5A–C).

**Figure 4:**
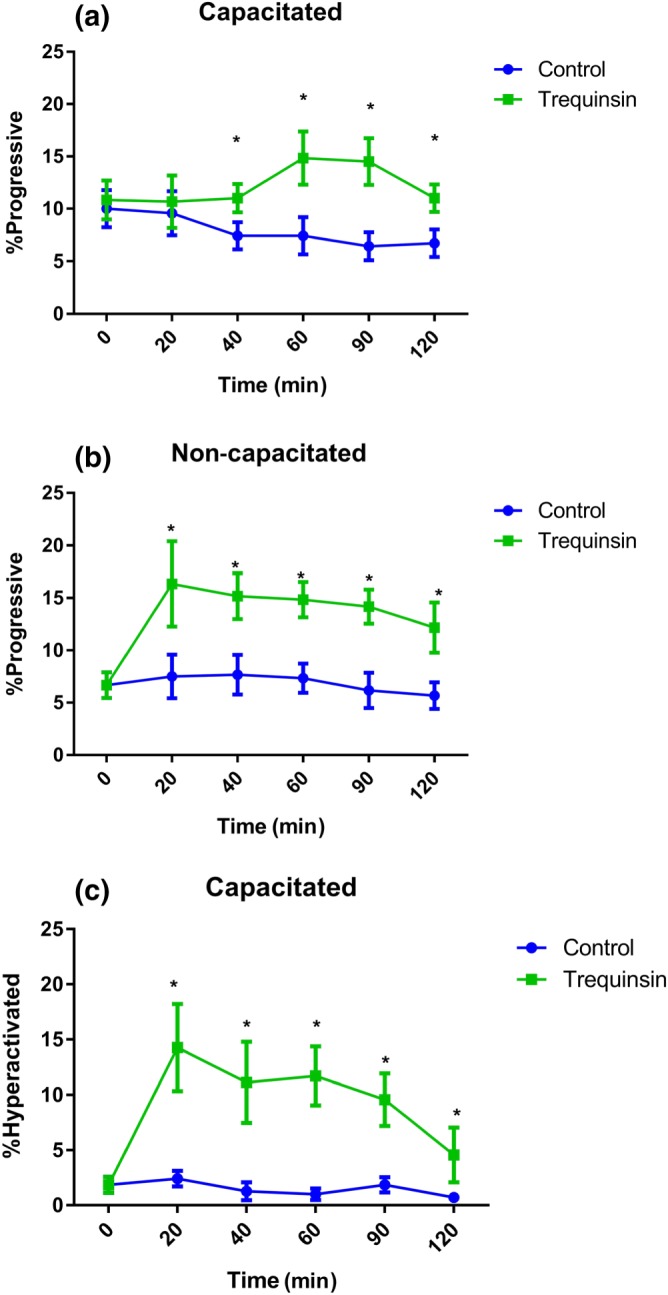
Effect of trequinsin on 40% DGC fraction (poor motility) donor sperm motility. Trequinsin significantly increased the percentage of progressively motile sperm in (a) capacitating (*n* = 5) and (b) non‐capacitating (*n* = 6) conditions. Hyperactivation was also significantly increased when sperm were incubated in (c) capacitating conditions (*n* = 8). *P<.05, significantly different compared to time 0; two‐way ANOVA with Sidak's multiple comparison analysis. Corresponding motility data can be found in Figure S6 [Corrections added on 27 December 2019, after first online publication: Figure 4 parts B and C have been relabelled.]

Cells with poor motility isolated from 40% fraction after DGC preparation of ejaculates from healthy volunteer sperm donors were initially used as a surrogate for patient sperm, as previously described (Tardif et al., 2014). In contrast to donor 80% fraction cells incubated in capacitating conditions, 40% fraction capacitated cells showed a trequinsin‐induced significant increase in PM 40 min after initial exposure, which was maintained for the duration of the assay period (Figure 4a). Although there was no effect on TM (Figure S6A), hyperactivation was also significantly increased, similar to donor 80% fraction cells (Figure 4b). Under non‐capacitating conditions, trequinsin significantly improved PM of 40% fraction sperm for the entire experimental period (Figure 4c) but had no effect on TM or hyperactivation (Figure S6B,C). The significant changes in motility seen in sperm from the 40% DGC fraction in capacitating conditions provided proof of concept that trequinsin may similarly boost sperm motility in poorly motile sperm from patients. To investigate this further, we assessed patient sperm motility over a 2‐hr period in response to treatment with trequinsin exposed to capacitating conditions.

#### 3.2.2 Patient sperm assessment

A total of 25 patients attending the ACU for routine andrology assessment, in vitro fertilisation, ICSI, and sperm study patients (Table 2) consented to provide their sample for research. Trequinsin increased the percentage of hyperactivated cells in the majority (88%) of capacitated patient sperm samples (22/25). TM and PM were unaffected by treatment with trequinsin in the majority of samples (18/25 for TM and 18/25 samples for PM). Of note, trequinsin did not alter motility parameters in two samples (R2946 and R2947) and negatively affected all motility parameters in only one sample (R2117).

**Table 2:** Effect of trequinsin on patient sperm motility

Patient ID	Initial WHO semen criteria assessment			Effect of trequinsin on patient sperm motility		
	Conc. (M·ml^−1^)	PM (%)	Classification	TM (%)	PM (%)	HA (%)
R2117	✓	✓	Normal	↓	↓	↓
R2926	✓	✓	Normal	—	—	↑
R2929	✓	✓	Normal	—	—	↑
R2937	✓	✓	Normal	—	↓	↑
R2939	✓	✓	Normal	—	—	↑
R2945	✓	✓	Normal	—	↓	↑
R2946	✓	✓	Normal	—	—	—
R2947	✓	✓	Normal	—	—	—
R2949	✓	✓	Normal	↑	↑	↑
R2951	✓	✓	Normal	—	—	↑
R2952	✓	✓	Normal	—	—	↑
R2919	✓	✓	Normal	—	—	↑
R2927	✓	×	Borderline	↑	—	↑
R2792	✓	×	Borderline	—	—	↑
R2931	✓	×	Borderline	↓	—	↑
R2935	✓	×	Borderline	—	—	↑
R2943	×	✓	Borderline	—	—	↑
R2950	✓	×	Borderline	↑	↑	↑
R2953	✓	×	Borderline	—	—	↑
R2971	✓	×	Borderline	—	—	↑
R2974	✓	×	Borderline	—	—	↑
R2976	×	×	Low	↑	↑	↑
R2340	×	×	Low	—	—	↑
R2730	×	×	Low	↑	↑	↑
R2938	×	×	Low	—	—	↑

*Note.* Summary of motility changes in patient samples (in vitro fertilisation, ICSI, and andrology) treated with 10‐μM trequinsin. The motility of 25 patient samples was assessed using CASA over a 2‐hr period at regular intervals (see Section 2), and an average for each parameter was taken overall. A minimum of 200 cells were counted at each time point. **↑**, significant increase; **—**, no change; **↓**, significant decrease. Significant means and *SD* (control vs. treatment at each time point) do not or do overlap for increase and decrease, respectively (TM, total motility; PM, progressive motility; HA, hyperactivated motility). Patient samples are categorised based on semen World Health Organization (WHO) parameters (see Section 2). ✓ represents a WHO guideline criterion met; × represents a criterion not meeting WHO guidelines.

### 3.3 Phase 3: Molecular actions of trequinsin

**Figure 5:**
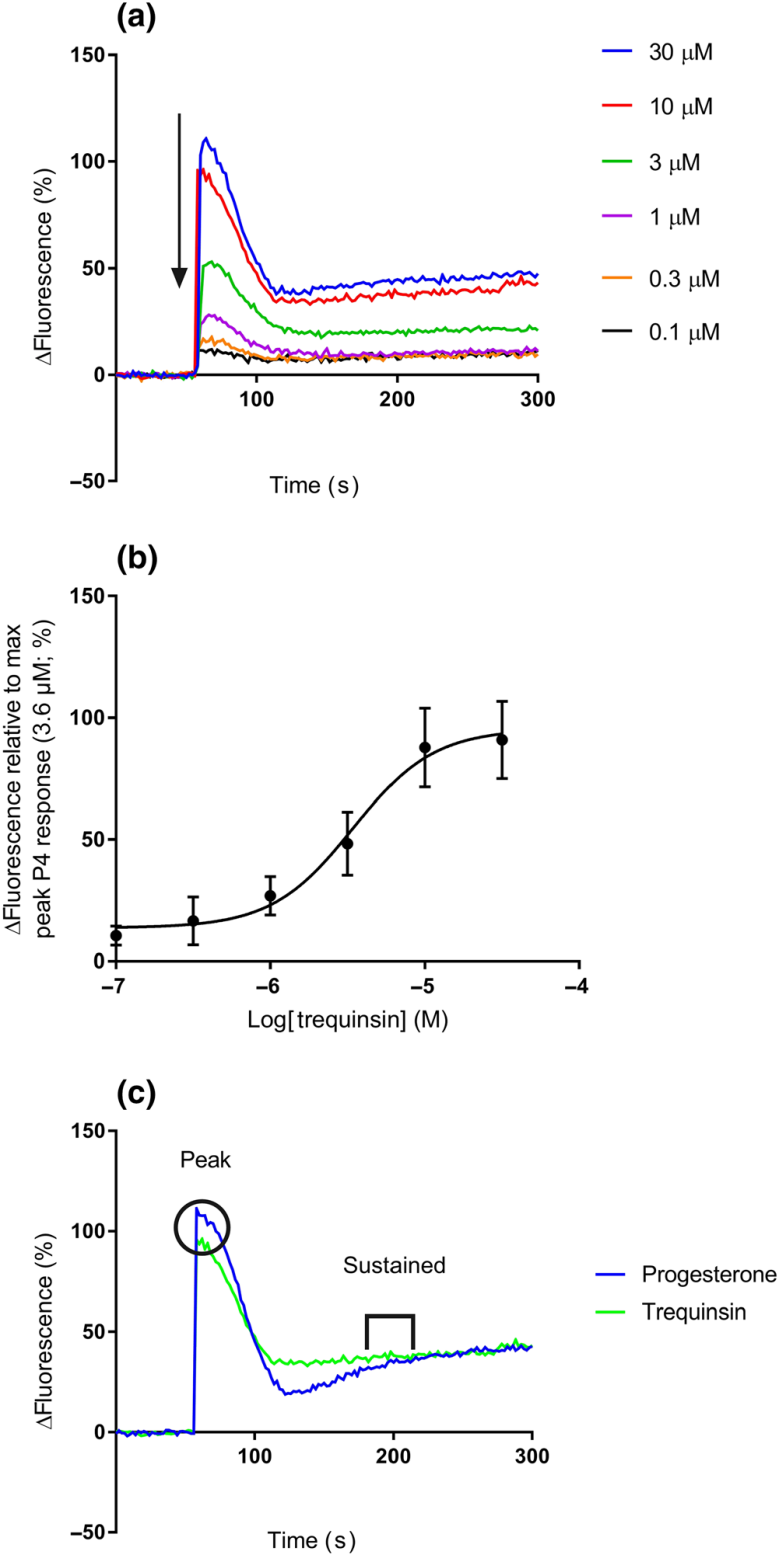
Effect of trequinsin on [Ca^2+^]_i_ in capacitated donor cells*.* (a) Mean dose response traces for trequinsin (0.1–30 μM). (b) Dose–response curve for trequinsin induced peak of [Ca^2+^]_i_ relative to progesterone (3.6 μM; EC_50_ = 3.43 μM [95% confidence limit: 2.19–5.82 μM]; *n* = 5) in 80% DGC fraction capacitated donor sperm. (c) Mean data set (*n* = 6) trace of 10‐μM trequinsin and 3.6‐μM progesterone [Ca^2+^]_i_ recording (↓ represents the addition of compounds, O highlights the peak, and П represents the sustained [between 180 and 200 s] fluorescent measured). Peak and sustained responses for progesterone and trequinsin are not significantly different

Elevated hyperactivation was the most consistent effect induced by trequinsin. Therefore, we subsequently explored the molecular actions of sperm incubated in capacitating conditions. We first analysed the trequinsin‐induced [Ca^2+^]_i_ increase from capacitated donor cell populations (80% DGC) normalised to a saturating concentration of progesterone (3.6 μM; to control for unwanted sources of variation). Trequinsin induced a concentration‐dependent increase in [Ca^2+^]_i_ (trequinsin‐induced peak EC_50_ = 3.43 μM [95% confidence limit: 2.19–5.82 μM]; Figure 5a,b), with an agonist profile analogous to progesterone. Interestingly, although the potency of progesterone is reported to be higher (progesterone peak = EC_50_ 33 nM; Strünker et al., 2011), the efficacy of 10‐μM trequinsin was equivalent to progesterone (Figure 5c).

**Figure 6:**
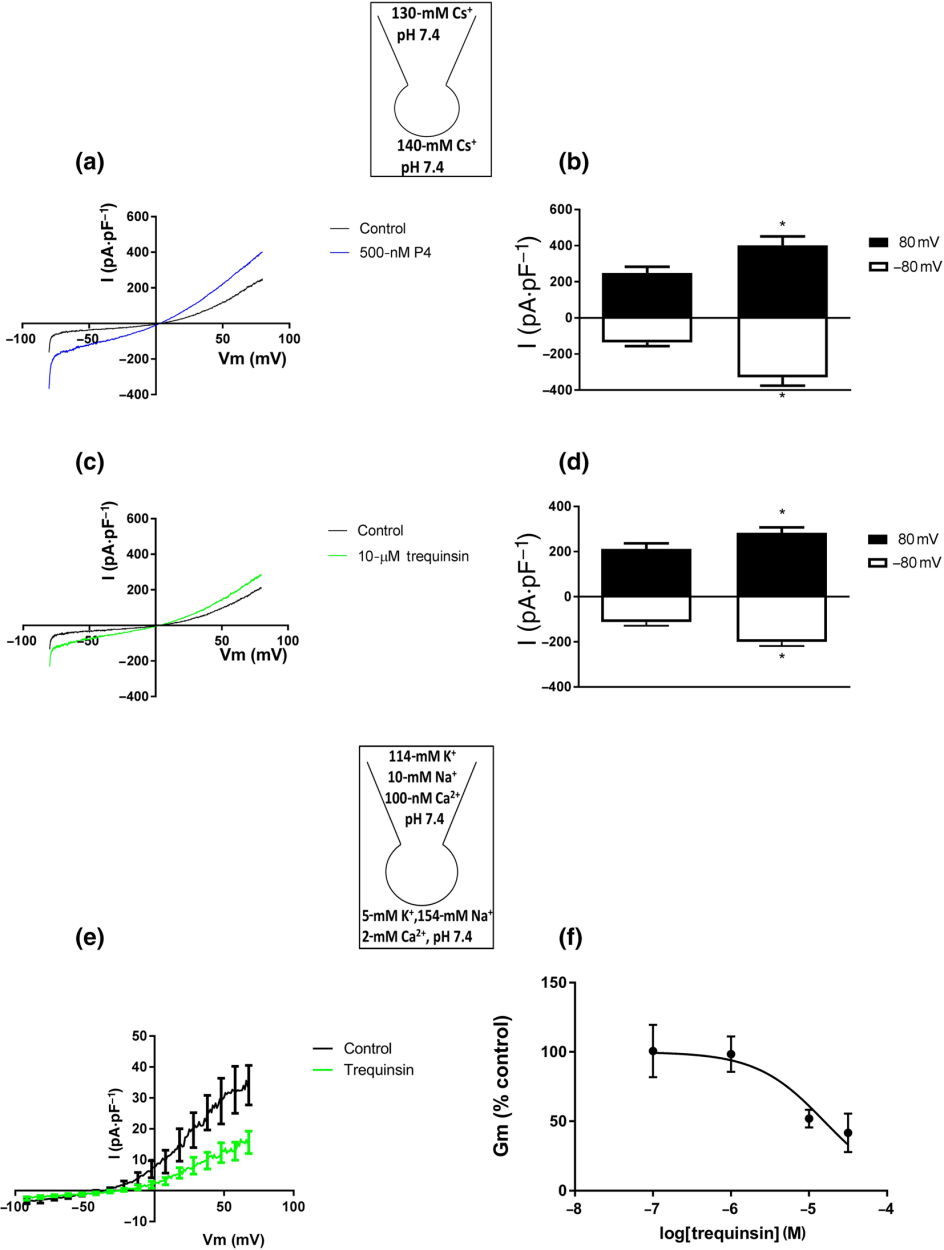
Patch clamp electrophysiology. (a, b) Inward CatSper‐mediated Cs^+^ currents (*n* = 6) in response to trequinsin (10 μM) were not significantly different to progesterone (P4) (c, d). *P<.05, significantly different from control response; two‐way ANOVA with Sidak's multiple comparison analysis. (e) I–V relationship showing the shift in reversal potential and Gm inhibitory effect caused by 10‐μM trequinsin (*n* = 7). (f) Dose–response curve for showing the partial inhibitory effect of trequinsin on Gm (*n* = 5). Patch clamp solution configurations are shown in insets. Donor sperm were from the 80% DGC fraction incubated in capacitating conditions

**Figure 7:**
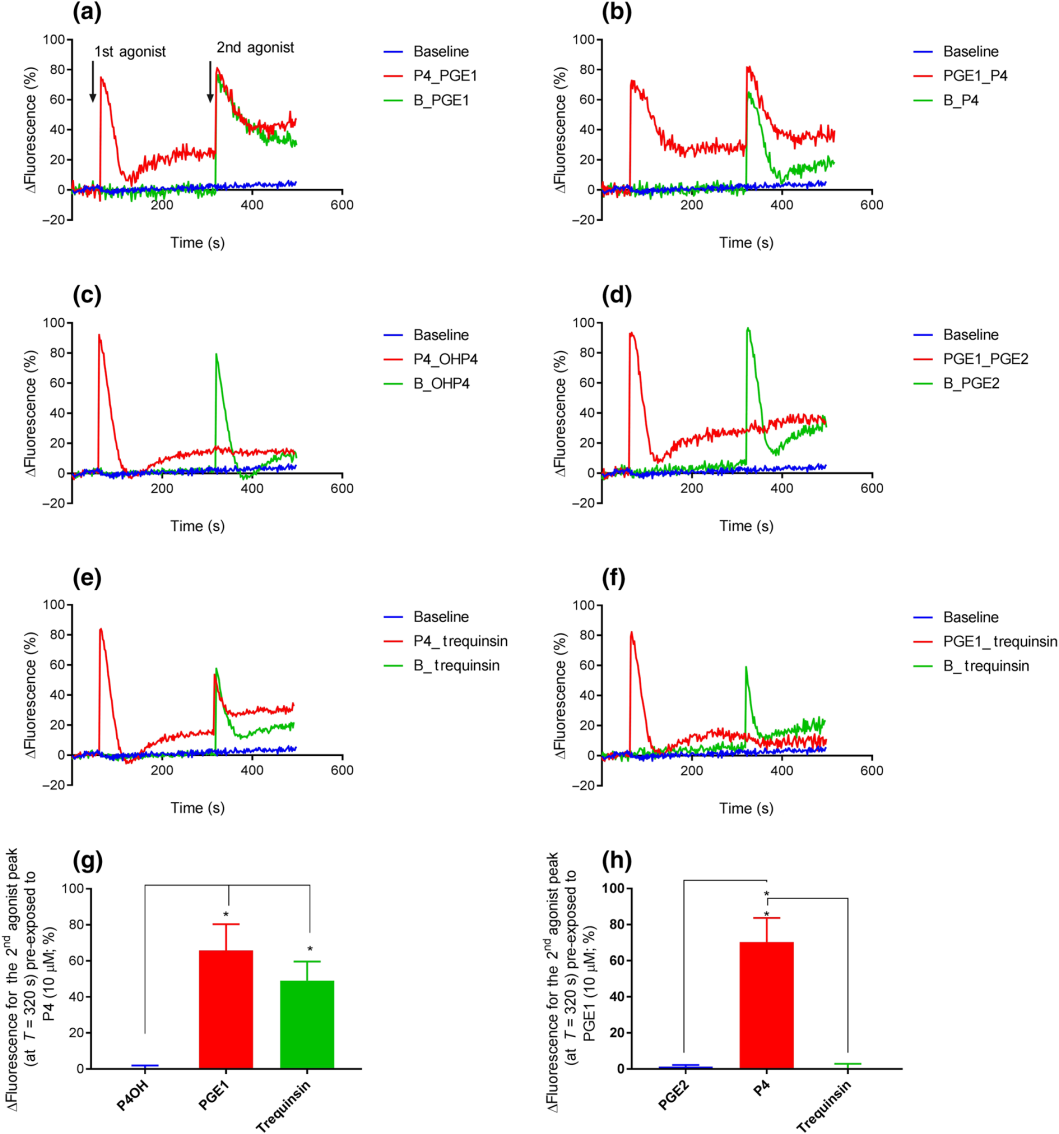
Examination of agonist cross‐desensitisation*.* Population average [Ca^2+^]_i_ trace using capacitated donor sperm from the 80% DGC fraction (*n* = 5) showing initial agonist addition of either a saturating concentration of 10‐μM progesterone (P4) (a, c, e) or 10‐μM PGE_1_ (b, d, f), followed by the second agonist addition. A baseline control shown in blue was included in each experiment and a blank (sEEBS, represented as “B”) followed by the addition of the second agonist green. Cross‐desensitisation experiments are shown in red. (g) Bar chart showing cell exposed to 10‐μM 17‐OH‐ progesterone (17OHP4) did not produce a significant Ca^2+^ response compared to that of PGE_1_ (10 μM) and trequinsin (10 μM). Effects of PGE_1_ and trequinsin were not significantly different. *P<.05, significantly different as indicated; two‐way ANOVA with Sidak's multiple comparison analysis. (h) Cells pre‐exposed to 10‐μM PGE_2_ had significantly lower Ca^2+^ responses (<2%) compared to progesterone exposure. Effects of PGE_2_ and trequinsin were not significantly different. *P<.05, significantly different as indicated; two‐way ANOVA with Sidak's multiple comparison analysis

**Figure 8:**
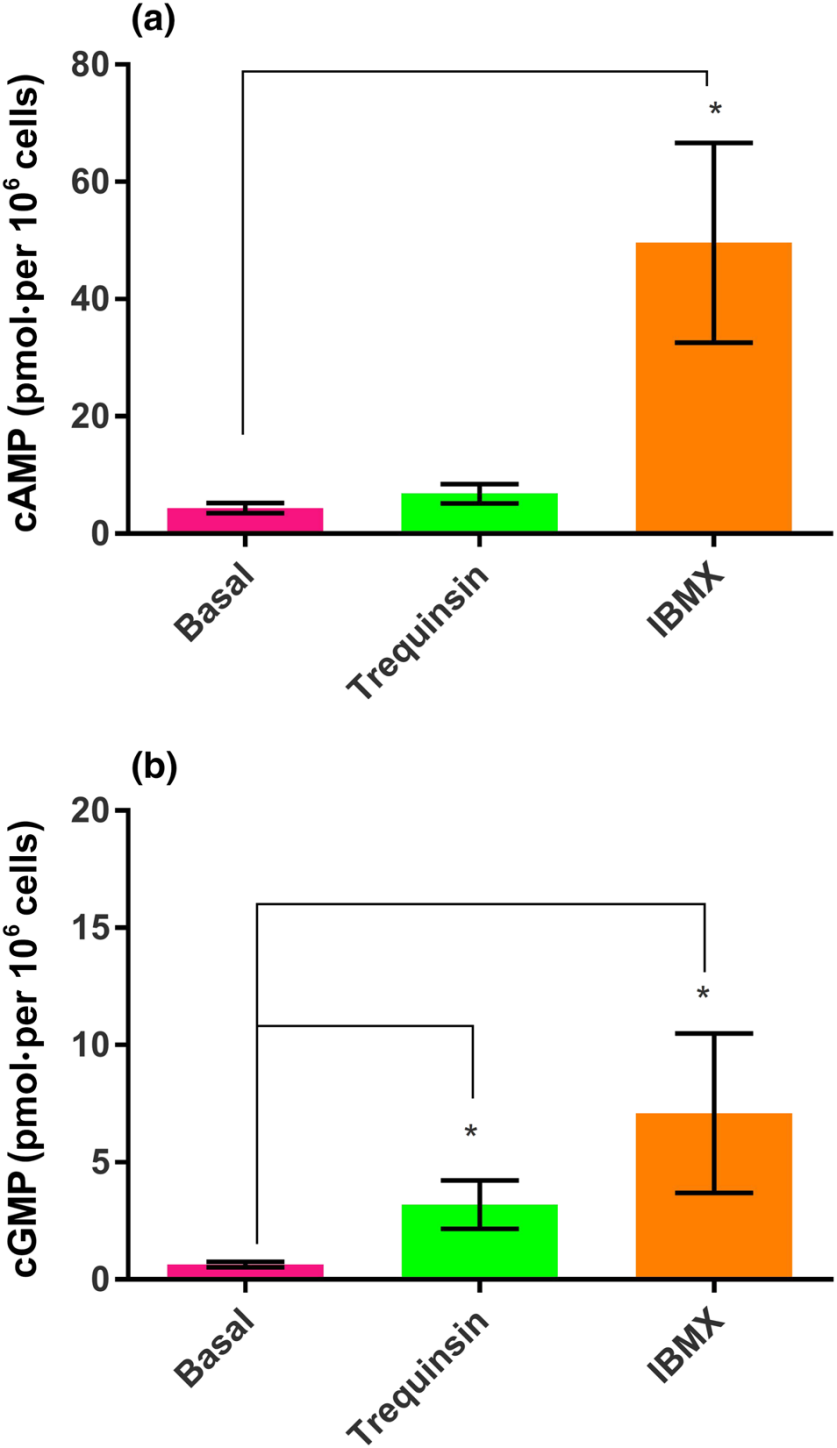
Measurement of cyclic nucleotide levels in capacitated 80% DGC fraction donor sperm using RP‐HPLC. (a) Trequinsin did not alter intracellular cAMP in comparison to control (cells +1% DMSO; *n* = 11). (b) Trequinsin significantly increased intracellular cGMP (*n* = 11). IBMX, a non‐specific PDEi, was used as a positive control. IBMX significantly increased both cAMP and cGMP (*n* = 11). *P<.05, significantly different as indicated; two‐way ANOVA with Sidak's multiple comparison analysis

CatSper is a ligand‐activated, pHi and voltage‐sensitive channel. Therefore, to investigate the mechanism by which trequinsin causes an increase in [Ca^2+^]_i_, we utilised whole‐cell patch clamp electrophysiology to examine the drug's ability to modulate ion channel function directly and monitored changes in pHi using the ratiometric dye 2′,7′‐bis(2‐carboxyethyl)‐5,6‐carboxyfluorescein. Predictably, trequinsin significantly potentiated inward and outward CatSper currents, to a degree not significantly different from progesterone (Figure 6a,b). However, trequinsin also had inhibitory activity on http://www.guidetopharmacology.org/GRAC/ObjectDisplayForward?objectId=387 function as 10‐μM trequinsin also significantly suppressed the membrane slope conductance of outward current (Figure 6e,f) causing a significant shift in reversal potential (control = −29.7 ± 6.6 mV, +10‐μM trequinsin = −17.4 ± 6.7 mV, Figure 6e). Trequinsin had no direct effect on pHi (Figure S7). PGE_1_ and progesterone activate CatSper through mechanisms that exhibit limited cross‐desensitisation (Brenker et al., 2018; Lishko et al., 2011; Miller et al., 2016a, 2016b; Schaefer et al., 1998; Shimizu et al., 1998; Strünker et al., 2011). Therefore, we exploited this phenomenon to investigate the mechanism of the trequinsin‐induced increase in [Ca^2+^]_i_. Pretreatment with progesterone caused desensitisation of the response to 17‐OH‐progesterone but not PGE_1_ or trequinsin (Figure 7). Fittingly, pretreatment with PGE_1_ caused desensitisation of the trequinsin, but not the progesterone response (Figure 7). Trequinsin is a potent PDE3i (Tinsley et al., 2009). PDE enzymes control the hydrolysis of cyclic nucleotides, specifically https://www.guidetopharmacology.org/GRAC/LigandDisplayForward?ligandId=2352 and https://www.guidetopharmacology.org/GRAC/LigandDisplayForward?ligandId=2347, both of which are substrates for PDE3 (Lefièvre et al., 2002). In contrast to the non‐specific PDEi IBMX, trequinsin did not significantly induce elevation of cAMP (Figure 8a). However, it induced a significant ~4‐fold increase of cGMP in capacitated cells (Figure 8b).

#### 3.3.1 Patient [Ca^2+^]_i_ profile in response to trequinsin

Given that poor motility and impaired fertilisation potential are associated with impaired CatSper function (Kelly et al., 2018), it is important to determine, where feasible, the functionality of CatSper in patient sperm. Sufficient spermatozoa were available in 9/25 patient samples to examine the ability of trequinsin to increase [Ca^2+^]_i_; 10‐μM trequinsin increased [Ca^2+^]_i_ as efficaciously as progesterone (Figure S8), indicating no fundamental abnormality in calcium signalling in these samples. Interestingly, trequinsin did not alter motility parameters of sperm from patient R2947 despite a robust increase (>50% ΔF in [Ca^2+^]_i_; Figure S10).

## 4 DISCUSSION

Male infertility is a significant health challenge that is estimated to affect one in 10 men (Datta et al., 2016). In up to 40% of these cases, the cause may be due to reduced sperm motility (asthenozoospermia; van der Steeg et al., 2011). However, as there are currently no licensed agents to treat infertile men, ICSI remains the only viable treatment option to ensure oocyte–spermatozoon interaction. A fundamental reason for the shortfall in progression in the field of male fertility therapeutics has been the lack of knowledge regarding a suitable molecular target in sperm, thereby limiting the opportunity for implementing drug discovery strategies (Barratt, De Jonge, & Sharpe, 2018; Hughes, Rees, Kalindjian, & Philpott, 2011). However, a wealth of studies now demonstrate that CatSper is a key determinant of sperm motility and fertilisation competence (Alasmari, Costello, et al., 2013; Brown et al., 2018; Kelly et al., 2018; Ren et al., 2001; Smith et al., 2013; Strünker et al., 2011; Williams et al., 2015) and therefore represents a plausible target for the development of novel therapeutics for male infertility. We have previously described a high‐throughput drug screening methodology in conjunction with relevant in vitro tests to identify compounds that increase functional sperm motility (Martins da Silva et al., 2017). While this study validated our drug discovery strategy, there continues to be a significant unmet clinical need to identify efficacious compounds that influence different forms of sperm motility and function. In this study, we utilised an HTS strategy to screen an in‐house drug discovery library and identified trequinsin hydrochloride, a putative selective PDE3i, which significantly increased [Ca^2+^]_i_.

CatSper is the primary calcium‐conducting plasma membrane ion channel in sperm that is activated by intracellular alkalinisation, membrane depolarisation, and physiological ligands such as progesterone and PGE_1_ (Singh & Rajender, 2015; Strünker et al., 2011; Tamburrino et al., 2014). It can also be manipulated by compounds, including endocrine disrupting chemicals, that may compromise sperm function (Schiffer et al., 2014; Tavares et al., 2013). Detailed analysis of the trequinsin‐induced [Ca^2+^]_i_ increase in cell populations showed that the kinetics of the response mirrored that of progesterone. However, while whole‐cell patch clamp electrophysiology confirmed the ability of trequinsin to potentiate CatSper currents to a degree not significantly different from progesterone, it was also able to suppress outward Gm. Progesterone is proposed to activate CatSper indirectly through stimulation of a plasma membrane lipid hydrolase ABHD2 which metabolises endogenous inhibitory http://www.guidetopharmacology.org/GRAC/FamilyDisplayForward?familyId=943 to cause channel opening. In contrast, PGE_1_ activates the channel directly (Miller et al., 2016a, 2016b). We exploited observations that these mechanisms exhibit limited cross‐desensitisation (Brenker et al., 2018; Lishko et al., 2011; Miller et al., 2016a, 2016b; Schaefer et al., 1998; Shimizu et al., 1998; Strünker et al., 2011) to show that the trequinsin cross‐desensitisation profile is indistinguishable from that of PGE_1_. As trequinsin did not alter pHi, we conclude that trequinsin increases [Ca^2+^]_i_ by a combination of direct activation of CatSper as well as by membrane potential depolarisation through a partial blocking effect on the sperm potassium channel. However, we cannot rule out additional direct actions on pathways that regulate intracellular stores (Correia, Michelangeli, & Publicover, 2015) or extracellular calcium entry (De Blas et al., 2009; De Toni et al., 2016; Kumar et al., 2016).

Trequinsin is a potent (subnanomolar IC_50_) inhibitor of recombinant PDE3 (Tinsley et al., 2009). As cyclic nucleotides are essential second messengers for sperm motility (Balbach, Beckert, Hansen, & Wachten, 2018; Jansen et al., 2015; Mukherjee et al., 2016), we utilised HPLC to measure cAMP and cGMP changes in sperm exposed to trequinsin and demonstrated that only cGMP was significantly increased. Given that PDE3 enzymes metabolise cAMP and cGMP (Ahmad, Degerman, & Manganiello, 2012), this result is surprising because pharmacological and immunological evidence supports the presence of PDE3 in human sperm, localised to the postacrosomal region of the head (Lefièvre et al., 2002). In contrast, PDE3 isoforms were not among the seven PDE enzymes identified in a study analysing the human sperm proteome (Wang et al., 2013). Therefore, our data may reflect the inhibitory activity of cGMP‐PDE, http://www.guidetopharmacology.org/GRAC/FamilyDisplayForward?familyId=260#1315. The notion of non‐selective PDE‐inhibitory activity of trequinsin is supported by the relatively high concentration that is required to increase HA. In fact, 10‐μM trequinsin is above the IC_50_ at the cAMP‐specific http://www.guidetopharmacology.org/GRAC/FamilyDisplayForward?familyId=260#1297 and http://www.guidetopharmacology.org/GRAC/ObjectDisplayForward?objectId=1300 and cGMP‐specific http://www.guidetopharmacology.org/GRAC/ObjectDisplayForward?objectId=1304 (Souness & Rao, 1997; Tinsley et al., 2009; Wunder, Gnoth, Geerts, & Barufe, 2009). However, proteomic data do not support their expression in human sperm (Wang et al., 2013). It is notable that micromolar concentrations of PDEi are generally required to induce improvements in human sperm motility (Alasmari et al., 2013.; Lefièvre et al., 2002; Maréchal et al., 2017; Tardif et al., 2014) but the reason for this is unknown.

Given that NO donor compounds can modify sperm kinematic parameters, including VCL and straight line velocity, it is entirely plausible that an effect of trequinsin on cGMP levels may contribute to the changes seen in VCL and straight line velocity (Figure S11; Miraglia et al., 2011). In further support for this mode of action, trequinsin increased the percentage of sperm exhibiting HA and penetration into viscous medium under capacitating conditions in all donor samples. Reassuringly, premature acrosome reaction was not induced in these samples; implying sperm‐zona pellucida binding would not be hindered.

As expected, the increase in hyperactivation was dependent upon cell capacitation status. Hyperactivation was unaltered in cells maintained in non‐capacitating conditions, despite trequinsin giving a robust [Ca^2+^]_i_ increase in these cells (Figure S9). Although trequinsin was highly effective at increasing hyperactivation in patient sperm samples incubated in capacitating conditions (22/25), two were unresponsive, and all motility parameters were reduced in one. The reason for this profile is unknown, but we could demonstrate that one unresponsive case (R2947) was not due to defective [Ca^2+^]_i_ signalling (Figure S10). Biological variability is certainly seen within human sperm populations. Indeed, not all patients respond to drugs, and this finding may not be uncommon (Alvarez et al., 2003; Moohan, Winston, & Lindsay, 1993).

Consequently, the same level of exposure to a drug, for example, trequinsin, may result in different levels of biological effects in individual patients. This is the key concept encompassed by the term “individualised medicine.” Determining the reasons for the biological variability seen in this and other studies (Martins da Silva et al., 2017) is an important consideration for future drug development and is dependent upon robust screening strategies and phenotypic assays to identify and treat specific molecular and functional impairment. Additionally, the development of multi‐target compounds could be advantageous. For example, it would be interesting to determine if trequinsin could restore the fertilising potential of sperm affected by CatSper and sperm potassium channel dysfunction (Brown et al., 2016; Kelly et al., 2018; Williams et al., 2015).

In summary, we have shown that trequinsin hydrochloride is an efficacious CatSper agonist that suppresses sperm potassium channel activity, elevates cGMP (but not cAMP), and induces similar kinetics of [Ca^2+^]_i_ increase as progesterone through a mechanism that cross‐desensitises with PGE_1_ . This novel pharmacological profile results in a phenotype of increased hyperactivation and penetration into viscous medium, which is relevant to sperm function required for natural conception. We conclude that the pharmacological profile of trequinsin in human sperm is unique in terms of effect on multiple key intracellular mediators that influence sperm function (Esposito et al., 2004; Hess et al., 2005; Martins da Silva et al., 2017; Tardif et al., 2014; Williams et al., 2015) and holds promise as a novel agent to treat male infertility.

## AUTHOR CONTRIBUTIONS

All authors were involved in the design of the study. S.M.d.S. obtained funding for the library compound high‐throughput screening and identification of trequinsin as well as the recruitment and consent of patients. S.M.d.S., A.G.H., D.W.G., C.L.R.B., and S.G.B. contributed to the study design. R.C.M. performed the majority of the experiments and analysis. S.G.B. conducted the flow cytometry and patch clamp experiments and data analysis. All authors contributed to the construction, writing, and editing of the manuscript. The initial and interim manuscript was drafted by R.C.M., S.G.B., and S.M.d.S., S.G.B and J.F. obtained the funding for the studentship for R.C.M. All Authors approved the final manuscript.

## CONFLICT OF INTEREST

The authors declare no conflicts of interest.

## DECLARATION OF TRANSPARENCY AND SCIENTIFIC RIGOUR

This Declaration acknowledges that this paper adheres to the principles for transparent reporting and scientific rigour of preclinical research as stated in the *BJP* guidelines for https://bpspubs.onlinelibrary.wiley.com/doi/full/10.1111/bph.14207, and as recommended by funding agencies, publishers, and other organisations engaged with supporting research.

## Supporting information



Figure S1:
Dose–response curve measuring change in intracellular‐calcium evoked by Trequinsin using Flexstation Assay. Dose–response curve showing the mean percentage change (Δ fluorescence) of [Ca^2+^]_i_ at varying doses of Trequinsin. 5 donor samples were assessed. Trequinsin caused a concentration‐dependent increase in [Ca^2+^]_i_ (EC_50_ = 6.4 μM (95% Cl: 4.1 μM to 9.9 μM).
Figure S2: Dose–response Evaluation of Trequinsin on Donor 80% Fraction (Capacitated) Sperm Cell Motility. Dose–response curve showing Δ of motility induced by Trequinsin relative to untreated sperm cells (basal). (A) Percentage of total motile cells (B) percentage of progressively motile cells and (C) percentage of hyperactivated cells (%HA) (*n* = 5) under capacitating conditions. Measurements were taken 20 min after exposure to Trequinsin.
Figure S3: Effect of Trequinsin on Donor 80% Fraction (Capacitated) Sperm Cell Motility. In cells exposed to capacitating conditions, Trequinsin did not significantly alter (A) total motility or (B) progressive motility (*n* = 7). For the same data set %HA was significantly increased in a subpopulation of cells (Fig 2).
Figure S4: Effect of Trequinsin on Acrosome Status. Trequinsin did not increase acrosome reaction in capacitated healthy donor sperm (*n* = 5) in comparison to control (untreated cells). In the presence of Ionophore A23187 (positive control), there was a significant increase in the presence of acrosome‐reacted cells in comparison to control conditions. A minimum of 10000 events per condition was recorded.
Figure S5: Effect of Trequinsin on Donor 80% Fraction (Non‐ Capacitated) Sperm Cell Motility. Under non‐capacitating conditions, Trequinsin did not have a significant effect on cell (A) Total motility (B) Progressive motility or (C) %HA for the entire 2 hour period (*n* = 7).
Figure S6: Effect of Trequinsin on Donor 40% DGC Fraction (poor motility) Sperm Cell Motility. Trequinsin did not have a significant effect on (A) total motility in those sperm placed in capacitating conditions (*n* = 6). Under non‐ capacitating conditions the effect of Trequinsin on (B) total motility (*n* = 8) and (C) %HA was not significant (n = 8).
Figure S7: Effect of Trequinsin on Sperm Cell [pH]i. BCECF was used to track changes in intracellular pH in capacitated donor sperm from the 80% DGC fraction. (A) The standard curve used for [pH]i calibration. (B) Table showing that Trequinsin did not significantly increase intracellular pH in comparison to basal pH. The weak base NH4Cl significantly increased intracellular pH (*n* = 5).
Figure S8: Effect of Trequinsin on Patient [Ca^2+^]_i_. [Ca^2+^]_i_ response to Trequinsin and P4 in patient sperm (*n* = 9). (A) Peak and (B) Sustained (180 – 200 s) [Ca^2+^]_i_ was not significantly different.
Figure S9: Effect of Trequinsin on peak [Ca^2+^]_i_ in Non‐Capacitated Donor Cells. [Ca^2+^]_i_ response to Trequinsin and P4 (*n* = 5). (A) Peak and (B) Sustained responses (180–200 s) of [Ca^2+^]_i_ were not significantly different. Donor sperm were from the 80% DGC fraction.
Figure S10: Trequinsin induced peak [Ca^2+^]_i_ in sperm from Patient R2947. (A) Peak and (B) Sustained (180–200 s) response of patient R2947 Trequinsin induced [Ca^2+^]_i_ in relation to the average donor (data from Fig 5) and average patient (data from Supplementary Fig 8) Trequinsin peak [Ca^2+^]_i_ response.
Figure S11: VSL and VCL kinetic parameters. On assessment of 80% fraction, capacitated donor samples (*n* = 7). Trequinsin significantly increased mean VSL and VCL parameters in the total cell population in comparison to control condition.
Figure S12: Graphical Summary of [Ca^2+^]_i_ elevating Compounds Identified in Screen of the Chemogenomics library*.* 27 US Food and Drug Administration approved active compounds were identified from the DDU Chemogenomics library screen following Flexstation assay testing and categorised based on their ability to increase [Ca^2+^]_i_ (low to high percentage increase relative to 10 μM P4 (positive control in red). Green indicates negative control (1% DMSO).
Table S1. Trequinsin Compound Information. Compound information supplied by Tocris Bioscience.
