# Supplementary material for: Novel pharmacological actions of trequinsin hydrochloride improve human sperm cell motility and function
Source: Br J Pharmacol. 2019 Dec 11;176(23):4521–36. doi: 10.1111/bph.14814 (PMC6932944; doi:10.1111/bph.14814)
Supplement: Supplementary file 1 — Figure S1: Dose–response curve measuring change in intracellular‐calcium evoked by Trequinsin using Flexstation Assay. Dose–response curve showing the mean percentage change (Δ fluorescence) of [Ca2+]i at varying doses of Trequinsin. 5 donor samples were assessed. Trequinsin caused a concentration‐dependent increase in [Ca2+]i (EC50 = 6.4 μM (95% Cl: 4.1 μM to 9.9 μM). Figure S2: Dose–response Evaluation of Trequinsin on Donor 80% Fraction (Capacitated) Sperm Cell Motility. Dose–response curve showing Δ of motility induced by Trequinsin relative to untreated sperm cells (basal). (A) Percentage of total motile cells (B) percentage of progressively motile cells and (C) percentage of hyperactivated cells (%HA) (n = 5) under capacitating conditions. Measurements were taken 20 min after exposure to Trequinsin. Figure S3: Effect of Trequinsin on Donor 80% Fraction (Capacitated) Sperm Cell Motility. In cells exposed to capacitating conditions, Trequinsin did not significantly alter (A) total motility or (B) progressive motility (n = 7). For the same data set %HA was significantly increased in a subpopulation of cells (Fig 2). Figure S4: Effect of Trequinsin on Acrosome Status. Trequinsin did not increase acrosome reaction in capacitated healthy donor sperm (n = 5) in comparison to control (untreated cells). In the presence of Ionophore A23187 (positive control), there was a significant increase in the presence of acrosome‐reacted cells in comparison to control conditions. A minimum of 10000 events per condition was recorded. Figure S5: Effect of Trequinsin on Donor 80% Fraction (Non‐ Capacitated) Sperm Cell Motility. Under non‐capacitating conditions, Trequinsin did not have a significant effect on cell (A) Total motility (B) Progressive motility or (C) %HA for the entire 2 hour period (n = 7). Figure S6: Effect of Trequinsin on Donor 40% DGC Fraction (poor motility) Sperm Cell Motility. Trequinsin did not have a significant effect on (A) total motility in those sperm plac [file BPH-176-4521-s001.pdf]

Supplementary Data

Supplementary Figure 1

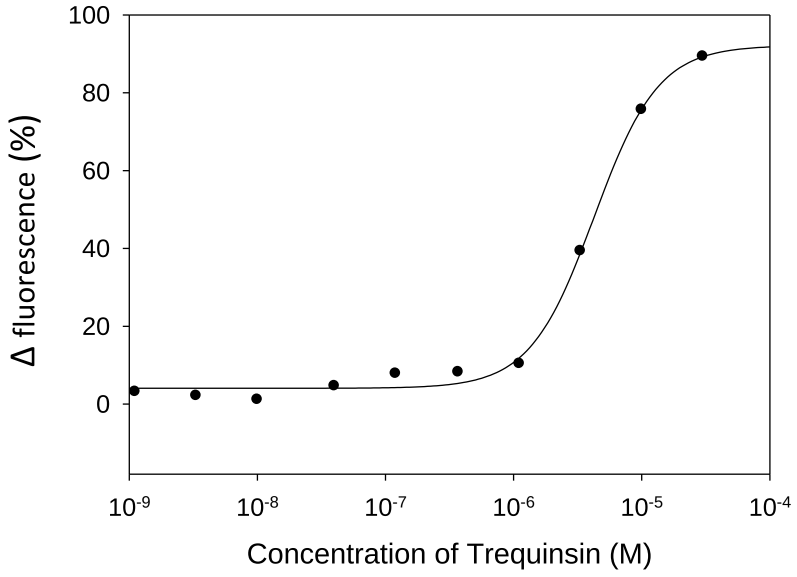

Supplementary Figure 2

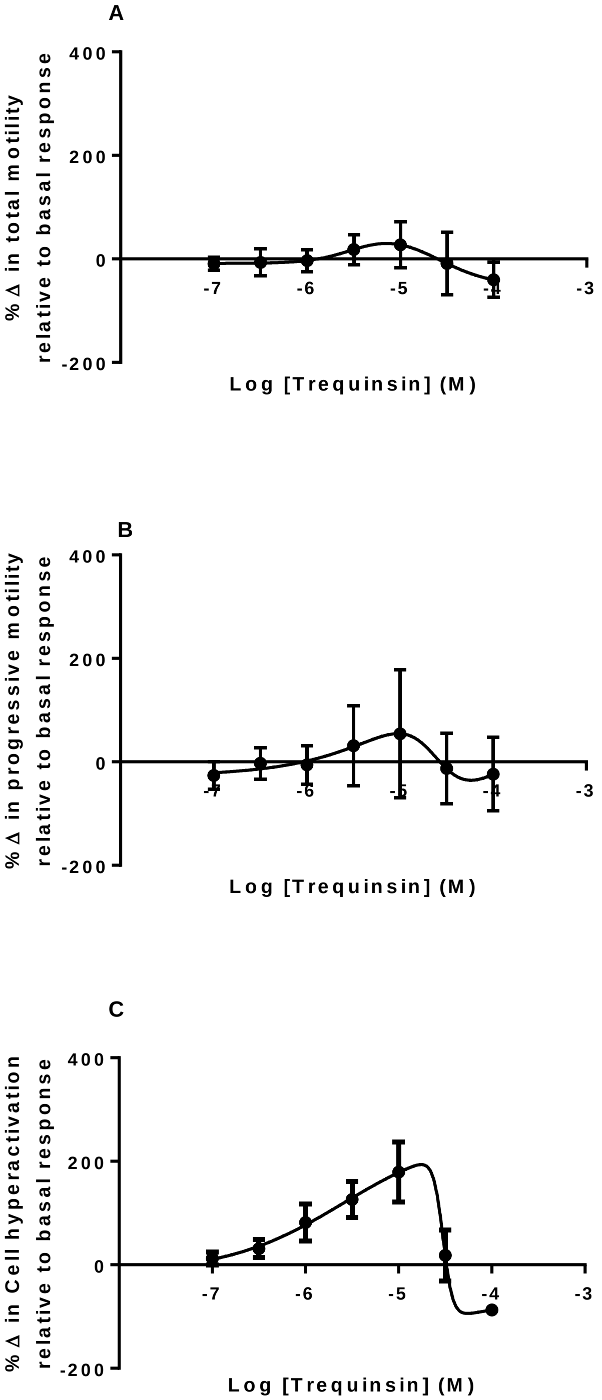

Supplementary Figure 3

A

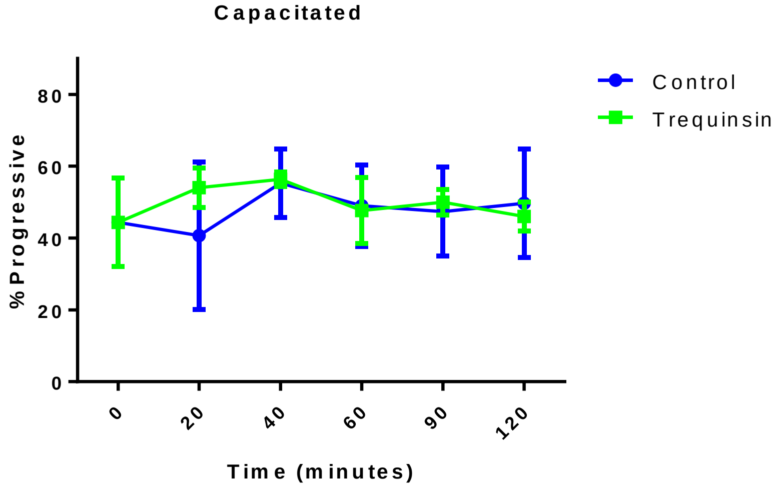

B

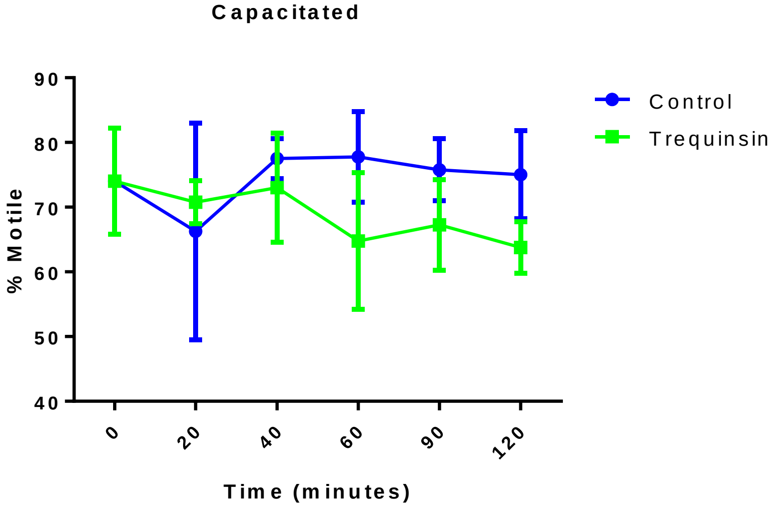

Supplementary Figure 4

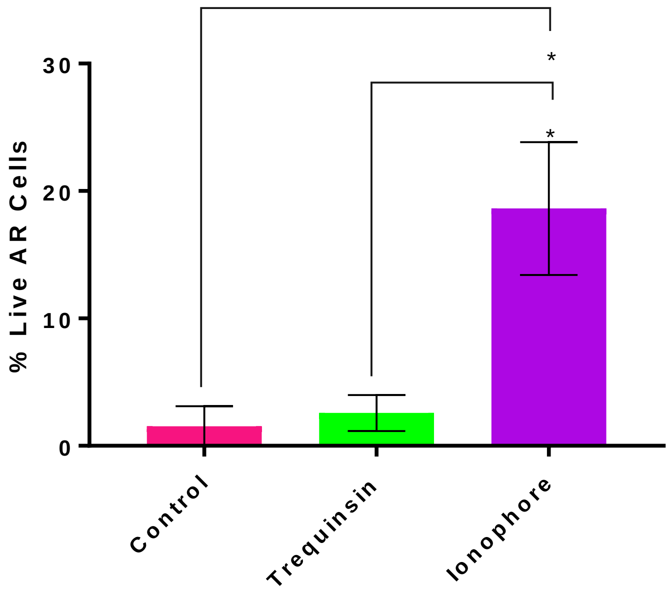

Supplementary Figure 5

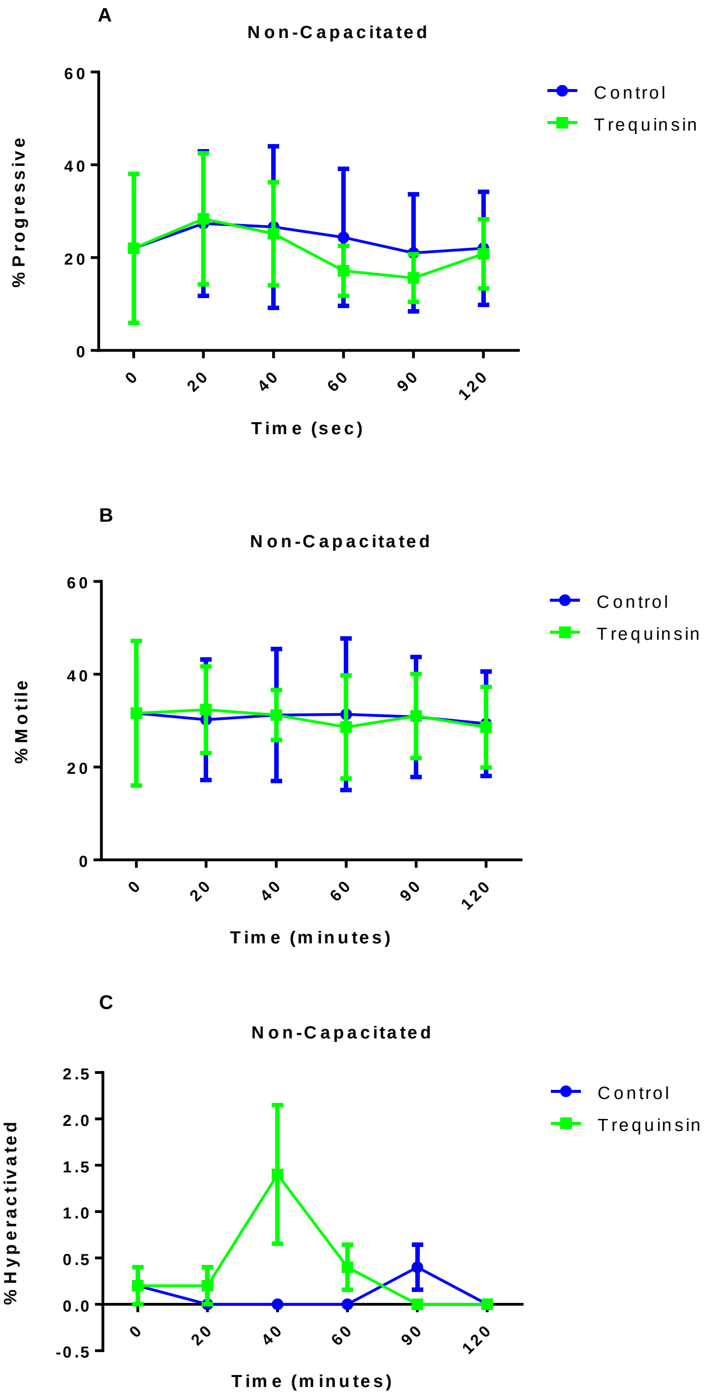

Supplementary Figure 6

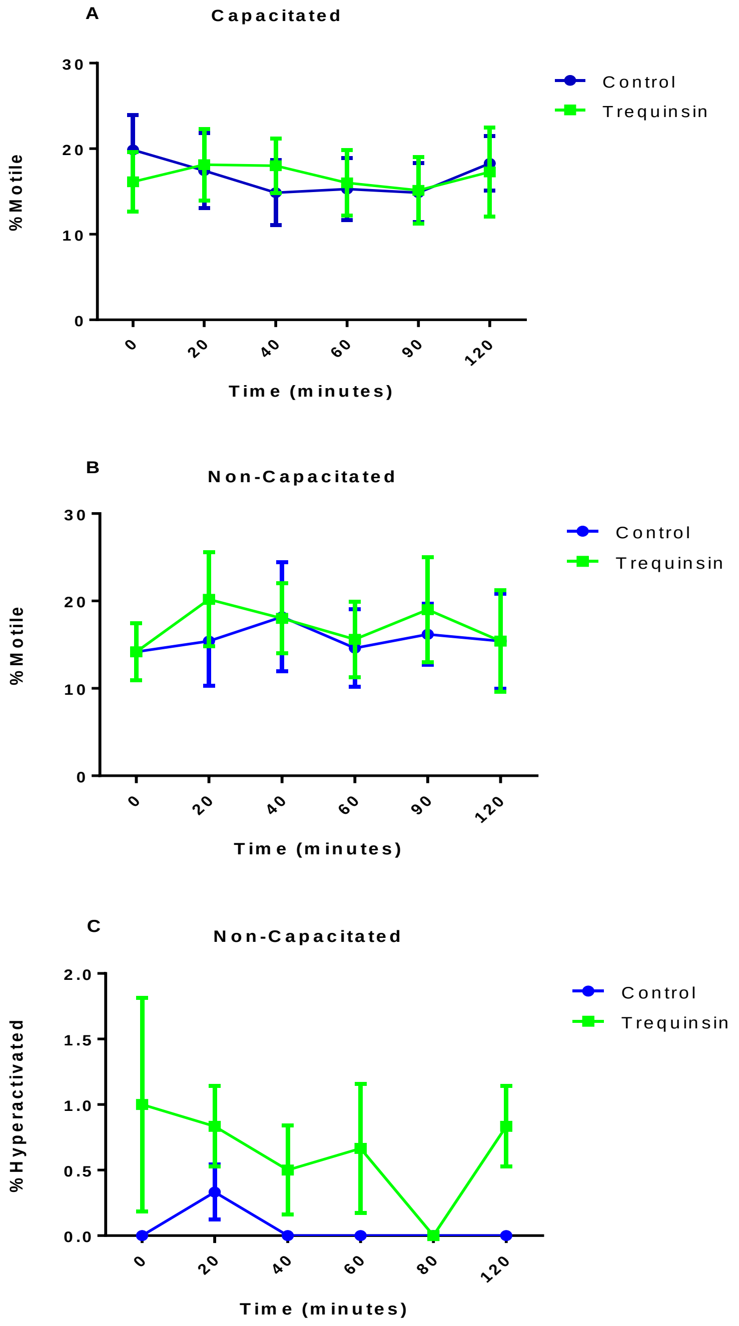

Supplementary Figure 7

A

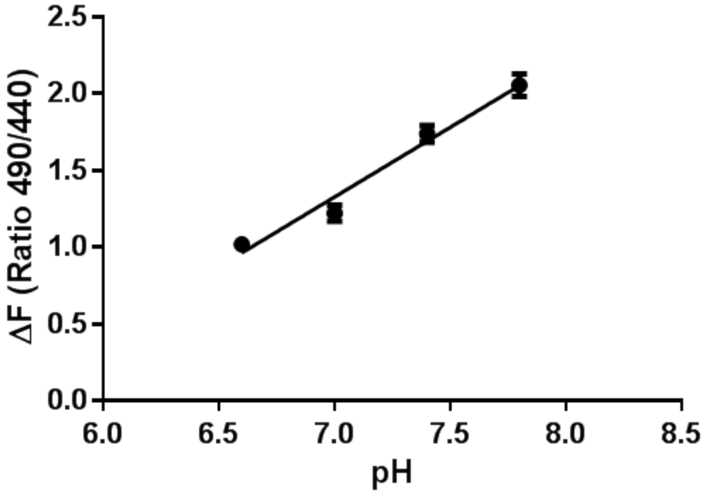

B

	pH	Ratio (490/440)	SD	SE
**Basal**	6.67	1.02	0.03	0.01
**Trequinsin**	6.72	1.07	0.06	0.03
**NH_4Cl**	7.04	1.36*	0.11	0.06

Supplementary Figure 8

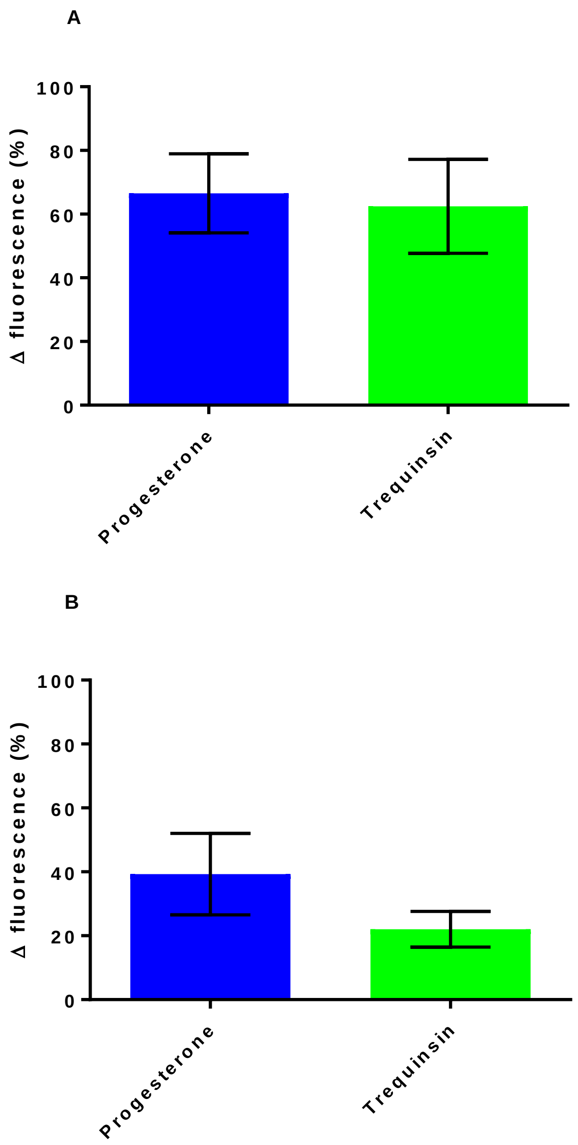

Supplementary Figure 9

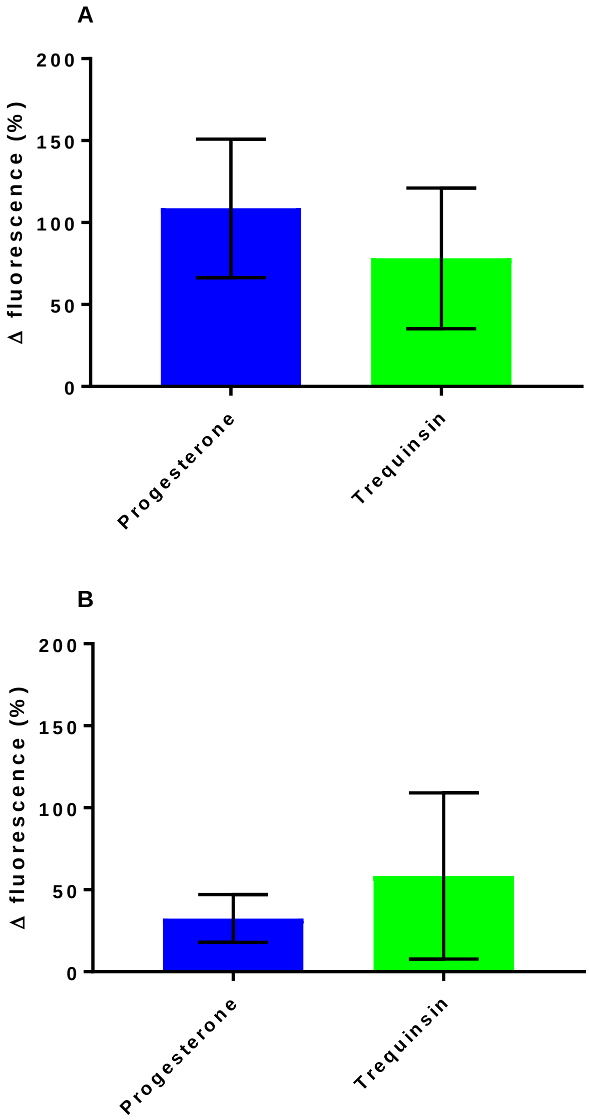

Supplementary Figure 10

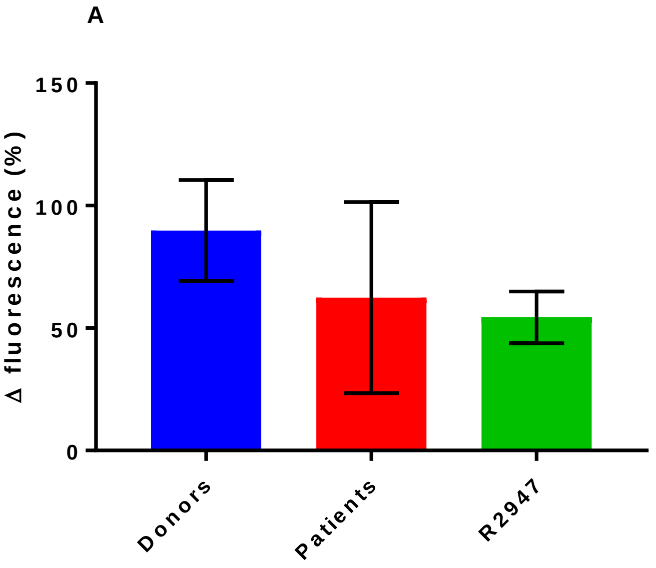

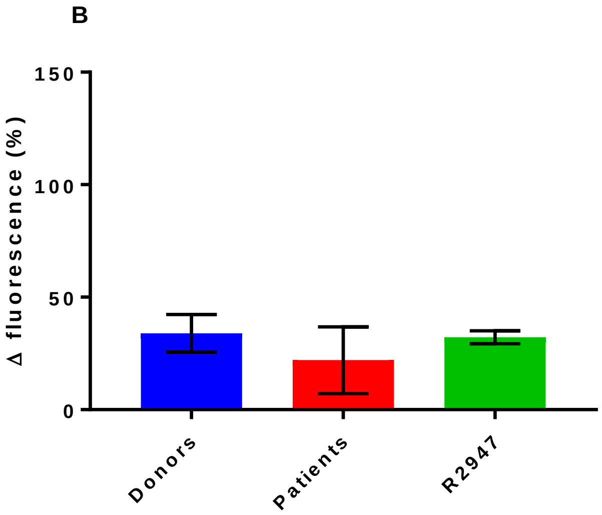

Supplementary Fig 11

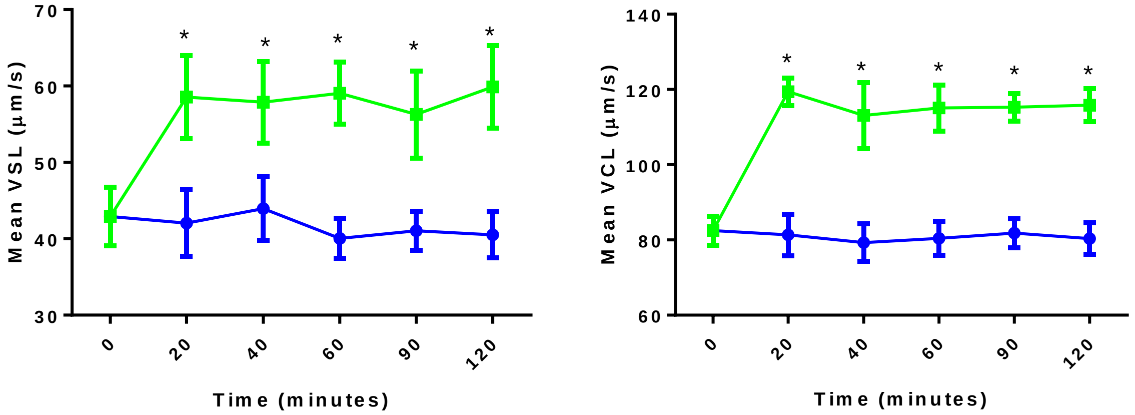

Supplementary Figure 12

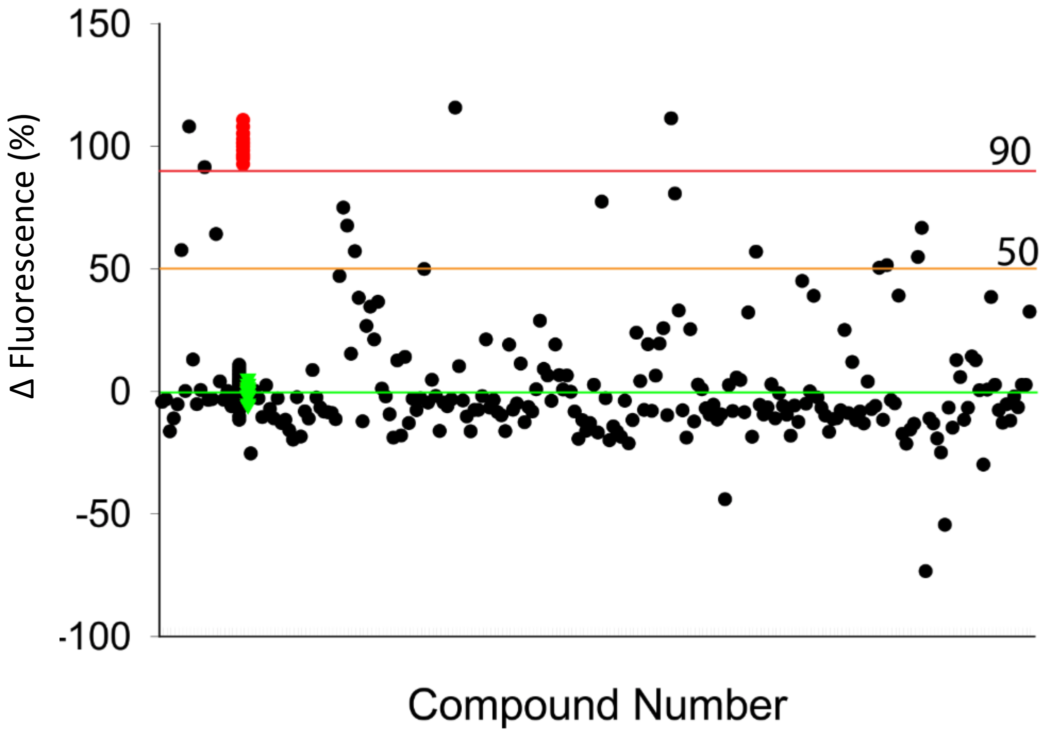

Supplementary Table 1. Trequinsin Compound Information. *Compound information supplied by Tocris Bioscience.*

**Product Name:**	Trequinsin hydrochloride
**CAS Number:**	78416-81-6
**IUPAC Name:**	2,3,6,7-Tetrahydro-9,10-dimethoxy-3-methyl-2-[(2,4,6-trimethylphenyl)imino]-4H-pyrimido[6,1-a]isoquinolin-4-one hydrochloride
**Description:**	Extremely potent inhibitor of cGMP-inhibited phosphodiesterase (PDE3; IC_{50} = 250 pM). Potently inhibits arachidonic acid induced aggregation of human platelets (IC_{50} = 50 pM). Orally active antihypertensive agent; reduces systemic blood pressure in both normotensive and hypertensive animal models. Physical and Chemical Properties: Batch Molecular Formula: C H N O .HCl Batch
**Molecular Weight:**	441.95
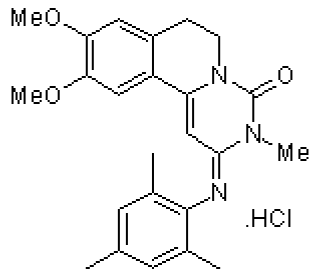	
